# The impact of selected methodological factors on data collection outcomes in observational studies of device-measured physical behaviour in adults: A systematic review

**DOI:** 10.1186/s12966-022-01388-9

**Published:** 2023-03-08

**Authors:** Richard M. Pulsford, Laura Brocklebank, Sally A. M. Fenton, Esmée Bakker, Gregore I. Mielke, Li-Tang Tsai, Andrew J. Atkin, Danielle L. Harvey, Joanna M. Blodgett, Matthew Ahmadi, Le Wei, Alex Rowlands, Aiden Doherty, Vegar Rangul, Annemarie Koster, Lauren B. Sherar, Andreas Holtermann, Mark Hamer, Emmanuel Stamatakis

**Affiliations:** 1 Faculty of Health and Life Sciences, University of Exeter, St Lukes Campus. EX12LU, Exeter, UK; 2 Department of Behavioural Science and Health, University College London, London, WC1E 7HB UK; 3 School of Sport, Exercise and Rehabilitation Sciences, University of Birmingham, Edgbaston, Birmingham, B15 2TT UK; 4 Radboud University Medical Centre, 6500 HB Nijmegen, The Netherlands; 5 School of Public Health, The University of Queensland, ST Lucia qld, Australia; 6 Center On Aging and Mobility, University Hospital Zurich, Zurich City Hospital – Waid and University of Zurich, Zurich , Switzerland; 7 Department of Aging Medicine and Aging Research, University Hospital Zurich and University of Zurich, Zurich, Switzerland; 8 Norwich Epidemiology Centre, University of East Anglia, Norwich, UK; 9 School of Health Sciences, Faculty of Medicine and Health Sciences, University of East Anglia, Norwich, NR47TJ UK; 10 Institute of Sport Exercise and Health, Division of Surgery and Interventional Science, University College London, London, W1T 7HA UK; 11 Charles Perkins Centre, School of Health Sciences, Faculty of Medicine and Health, The University of Sydney, Sydney, NSW Australia; 12 Diabetes Research Centre, University of Leicester, Leicester General Hospital, Gwendolen Road, Leicester, LE5 4PW UK; 13 NIHR Leicester Biomedical Research Centre, University of Leicester, Leicester, UK; 14 Alliance for Research in Exercise, Nutrition and Activity (ARENA), Division of Health Sciences, Sansom Institute for Health Research, University of South Australia, Adelaide, Australia; 15 Big Data Institute, Department of Population Health, University of Oxford, Oxford, OX3 7LF UK; 16 Department of Public Health and Nursing, HUNT Research Centre, Norwegian University of Science and Technology, Levanger, Norway; 17 Department of Social Medicine, CAPHRI, Care and Public Health Research Institute, Maastricht University, Maastricht, The Netherlands; 18 School of Sport, Exercise and Health Sciences, Loughborough University, Loughborough, LE113TU UK; 19 National Research Centre for the Working Environment, Copenhagen, Denmark

**Keywords:** Accelerometers, Physical Behaviour, Physical Activity, Sedentary Behaviour, Health, Measurement, Methods, Epidemiology, Observational Studies, Recruitment, Adherence

## Abstract

**Background:**

Accelerometer measures of physical behaviours (physical activity, sedentary behaviour and sleep) in observational studies offer detailed insight into associations with health and disease. Maximising recruitment and accelerometer wear, and minimising data loss remain key challenges. How varying methods used to collect accelerometer data influence data collection outcomes is poorly understood. We examined the influence of accelerometer placement and other methodological factors on participant recruitment, adherence and data loss in observational studies of adult physical behaviours.

**Methods:**

The review was in accordance with the Preferred Reporting Items for Systematic Reviews and Meta Analyses (PRISMA). Observational studies of adults including accelerometer measurement of physical behaviours were identified using database (MEDLINE (Ovid), Embase, PsychINFO, Health Management Information Consortium, Web of Science, SPORTDiscus and Cumulative Index to Nursing & Allied Health Literature) and supplementary searches to May 2022. Information regarding study design, accelerometer data collection methods and outcomes were extracted for each accelerometer measurement (study wave). Random effects meta-analyses and narrative syntheses were used to examine associations of methodological factors with participant recruitment, adherence and data loss.

**Results:**

123 accelerometer data collection waves were identified from 95 studies (92.5% from high-income countries). In-person distribution of accelerometers was associated with a greater proportion of invited participants consenting to wear an accelerometer (+ 30% [95% CI 18%, 42%] compared to postal distribution), and adhering to minimum wear criteria (+ 15% [4%, 25%]). The proportion of participants meeting minimum wear criteria was higher when accelerometers were worn at the wrist (+ 14% [ 5%, 23%]) compared to waist. Daily wear-time tended to be higher in studies using wrist-worn accelerometers compared to other wear locations. Reporting of information regarding data collection was inconsistent.

**Conclusion:**

Methodological decisions including accelerometer wear-location and method of distribution may influence important data collection outcomes including recruitment and accelerometer wear-time. Consistent and comprehensive reporting of accelerometer data collection methods and outcomes is needed to support development of future studies and international consortia. Review supported by the British Heart Foundation (SP/F/20/150002) and registered (Prospero CRD42020213465).

**Supplementary Information:**

The online version contains supplementary material available at 10.1186/s12966-022-01388-9.

## Background

Observational studies make a substantial contribution to our understanding of human behaviour, health and disease trajectories across the life-course, and in doing so inform developments in public health and healthcare policy and practice [1]. Evidence for physical activity (PA) as an important component of lifelong health has been established over more than 60 years of epidemiological research [2]. Those who are physically active have better cardiometabolic risk profiles, lower risk of non-communicable diseases [3], maintain better physical and mental function in aging, and live longer [4, 5] than those who are less active. More recent epidemiological evidence for associations of sedentary behaviours (SB) [6, 7] and sleep [8, 9] with health have expanded the previous PA focused paradigm to encompass ‘physical behaviour’ as a whole, where PA, SB and sleep are examined as interactive component behaviours within a 24-h cycle [10–12].

Understanding of associations between physical behaviours and health has improved substantially with the advent of wearable accelerometer-based approaches to quantifying behaviour in free-living at a population level [13]. Limitations in self-reported questionnaire-based measurements hinder research into physical behaviours in epidemiological research. Ongoing advancements in the use of body-worn movement sensors continue to change this landscape [14]. It is more than 30 years since accelerometers, were first used in physical behaviour research [15]. Developments in hardware, software, data processing and analysis now provide unprecedented insights into the frequency, intensity, duration and temporal distribution of human movements, postural classification and activity type [16, 17].

While accelerometers are now widely used in large-scale population research [18, 19] a key challenge remains regarding how to maximise the valid data collected in samples that represent the target population [1]. To continue to develop our understanding of associations between behaviours and health, it is imperative that we investigate how physical behaviours accumulate in varying patterns and contexts over complete 24-h periods [20]. Incomplete data limits our understanding of how and when behaviours occur and interact [21]. Inadequate recruitment, attrition, poor adherence to measurement protocols and data loss may threaten internal validity [22], introduce sampling bias [23] or otherwise undermine the utility of the behavioural data to address population health questions.

Decisions on the placement of the device (e.g., waist, wrist, thigh or at multiple locations) determines many aspects of the accelerometry methodology, including wear instructions, data cleaning, processing into meaningful variables (e.g., intensity, type) and analyses. The choice of placement may reflect research objectives or practical considerations, including (but not limited to) cost, device availability and participant burden. For example, participants’ acceptability and the practicality of different wear locations and associated wear protocols may vary between and within populations, environments and contexts, although it is not clear how this may differentially impact recruitment and adherence [24]. In addition, the impact on the relative success of data collection of practical factors such as study design and size/geographical coverage, how accelerometers are distributed and returned, and contact with participants during measurement, is unknown [25].

This review aims to systematically examine influence of device placement and other methodological factors on participant recruitment, adherence and data loss in observational studies of physical behaviours.

## Methods

This review was reported in line with the Preferred Reporting Items for Systematic reviews and Meta-Analyses (PRISMA) [26]. A protocol for the review was registered in Prospero (registration number CRD42020213465).

### Identification of observational studies

This review focused on the measurement of physical behaviours in observational (longitudinal, cohort or cross-sectional) studies. The unit of analysis for this review was study wave (sweep), i.e., each period of measurement of physical behaviours using accelerometers (as methods and data collection outcomes may vary between waves from the same study). In the first instance, observational studies were identified through systematic database searches for peer-reviewed journal articles describing their findings. Observational studies were included if the following criteria were met:

1) accelerometer-based measurement of physical behaviours (physical activity, sedentary behaviour, or posture including extrapolations of sleep).

2) observational studies that are either longitudinal (baseline measurement plus at least one repeat measurement), prospective (studies that implemented accelerometery measures once, and followed them up via linkage records), or cross-sectional studies (studies that measured participants once, without a repeat measurement or linkage) designed to cover national or regional populations.

3) adult participants (aged ≥ 18 years) either exclusively or reported separately from data on children or adolescents.

Studies were excluded if they reported data on children and adolescents only or reported data on cohorts of participants recruited due to the presence of or expected progression to a clinical condition. Searches were limited to articles published in English in peer-reviewed scientific journals, and were not limited by publication date.

Literature searches were conducted in March 2020, and updated in May 2022 using the following databases: MEDLINE (Ovid) 1946 to present and supplemented with MEDLINE (Ovid) ‘in-process’ and other non-indexed citations, Embase, PsychINFO, Health Management Information Consortium, Web of Science Core collection (including conference proceedings citation index, and emerging sources citation index), SPORTDiscus through EBSCOhost, and CINAHL (Cumulative Index to Nursing & Allied Health Literature) through EBSCOhost.

A base search strategy was developed in MEDLINE (Ovid) (Supplementary Figure S1) and search syntaxes altered accordingly for other databases. The search strategy included title, abstract and subject word searches for Medical Subheading (MeSH) terms relating to physical behaviour, physical activity, sedentary behaviour, objective measurement of movement (accelerometer, accelerometery, motion sensor, device) and terms denoting cross-sectional and prospective observational studies. Supplementary searches were completed through bibliographic screening, forward and backward citation searches of articles, and correspondence with experts in the field.

Following the removal of duplicates, titles and abstracts of all articles were independently screened by two reviewers (AA, MA, EB, JMB, LB, SF, DH, GM, RP, LT, LW) to assess whether the study described by the article was eligible for inclusion. Disagreement between reviewers as to the inclusion of a study was resolved in consultation with a third arbiter. Full-texts of included articles were then reviewed to assess the eligibility of the studies they describe.

### Data extraction

Information pertaining to the measurement of physical behaviours using accelerometers in every wave of each included study was identified and extracted using the following process:

1) Review of original articles returned in the literature searches and selected for inclusion.

2) Review of publicly available materials pertaining to each study.

3) Email requests to principal investigators and/or authors identified from published articles to provide any information not obtained in 1 and 2.

Using the above process, we collated information on the study characteristics and methods described in Table 1. Data extraction was piloted in eight cohort studies, leading to protocol revisions when necessary to ensure a consistent approach for all studies. In the case of longitudinal cohorts (multiple waves of accelerometer assessment), or cross-sectional studies with multiple independent samples, the above information was extracted separately for each study wave. Where studies employed multiple devices information was extracted for each device where possible. Where necessary extracted information was sent by the review team to a Principal or other Investigator from each study for final verification prior to data synthesis and analysis.

**Table 1:** Study characteristics extracted for each observational study wave

Study variable	Description
Descriptive information	
Study name	The given name of the cohort study and study acronym
Year of measurement	The year in which the accelerometer measurement began
Methods employed for collection of data on physical behaviours	
Accelerometer used	The accelerometer device chosen for data collection (manufacturer make and model)
Accelerometer wear location	The body location at which the accelerometer was worn
Accelerometer distribution method	The method by which participants received an accelerometer
Accelerometer return method	The method by which accelerometers were returned to investigators following measurement
Accelerometer wear instructions	The time period over which participants were requested to wear an accelerometer
Participant follow-up	Whether participants were contacted during the measurement period
Minimum valid wear criteria	The minimum amount of accelerometer wear required for a data file to be included in analyses
Outcomes from measurement of physical behaviours	
N Invited	The number of participants invited to wear an accelerometer
N Consented	The number of participants invited to wear an accelerometer who consented
N Devices lost	The number of accelerometers lost during measurement
Average daily valid accelerometer wear	Average daily valid accelerometer wear reported in hours per day (or calculated from reported values if possible)
N adhered	The number of people who met the minimum wear criteria and were included in analyses
Response bias	Reported demographic differences between those meeting wear criteria and those invited or the original study sample
Adverse events	The number and description of any adverse events relating to accelerometer wear

### Data synthesis

Synthesis of extracted data from each study wave was conducted using a hybrid approach consisting of meta-analyses (where data allowed) and narrative synthesis to examine consent, data loss, accelerometer wear, response bias, and reported adverse events.

### Meta analyses

Random effects meta-analyses (to allow for both within and between study variance) and meta-regression were conducted and reported in line with the PRISMA framework. We conducted meta-analyses of proportions of 1) invited participants that consented to wear an accelerometer, 2) participants that met minimum valid wear criteria, and 3) devices lost during measurement according to established best practice for systematic reviews of proportional data [27–29]. Proportions were initially transformed and within study variance computed using the Freeman-Tukey Double-Arcsine method [30, 31] in order to account for the skewed distribution of proportions, and to prevent the allocation of unduly large study weights to studies with proportions close to 0 or 1 [29].

Statistical heterogeneity between studies was assessed using the Cochrane Q test and I^2^ statistics [29, 32]. A ‘leave-one-out’ sensitivity analysis was completed to explore the influence of individual studies on overall pooled effect by removing one at a time from pooled analyses, and additionally for any statistical outliers.

Univariate and multivariable meta-regressions were used to investigate sources of between study variance in the three outcomes examined. Potential effect modifiers were: i) accelerometer wear location (classified as waist, thigh, wrist, a combined category for studies employing devices placed at the arm, chest, and back, or employing multiple sensors), ii) study design (prospective or cross-sectional), iii) sample size (number of participants who consented to wear a device separated into in quartiles: Q1 *n* < 745, Q2 *n *= 745–1514, Q3 *n* = 1515–3822, Q4 *n* > 3822), iv) study age (years since conduct of accelerometer data collection), v) accelerometer distribution method and (vi) return methods (in-person, post, or other), and vii) whether participants were contacted during the measurement period (yes or no).

Funnel plots and Egger’s test were used to explore potential reporting bias. Sensitivity analyses were conducted following exclusion of repeated measures of the same participants from within prospective cohort studies or with multiple devices in order to account for the possible effect of correlation between repeated measures. Analyses were conducted using the MetaProp [29] and MetaReg packages in Stata version 17 (Stata Corp LLC, College Station, TX, US).

### Narrative synthesis

Where the extracted data were not conducive to meta-analysis (i.e. accelerometer wear instructions, minimum wear criteria, and average daily accelerometer wear, response bias, and adverse events reported), this information was synthesised narratively in accordance with recent best practice guidelines [33] and reported in line with the PRISMA extension for systematic reviews without meta-analysis (SWiM) [34]. Studies were initially grouped according to accelerometer wear location for comparison of descriptive information including the range and frequency of different methodological practices and measures of central tendency and dispersion for continuous outcomes.

## Results

### Literature searches and cohort study characteristics

**Fig. 1:**
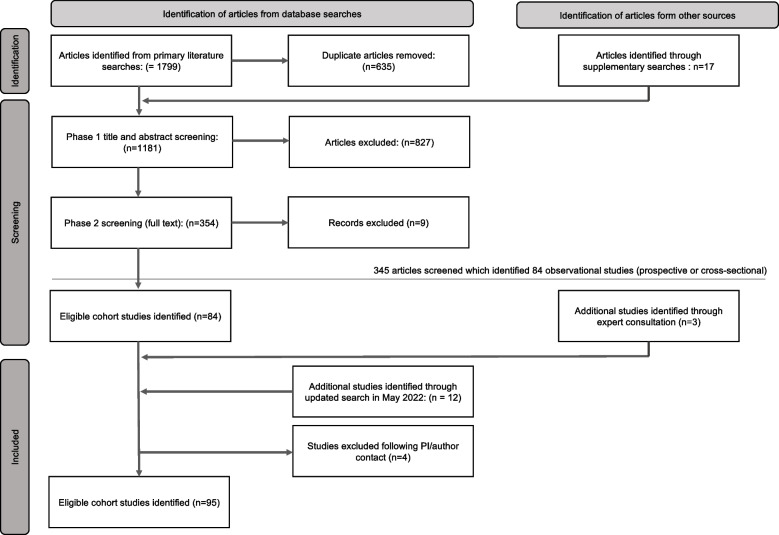
Adapted PRISMA flow diagram. Overview of the process for identification and selection of study waves

**Fig. 2:**
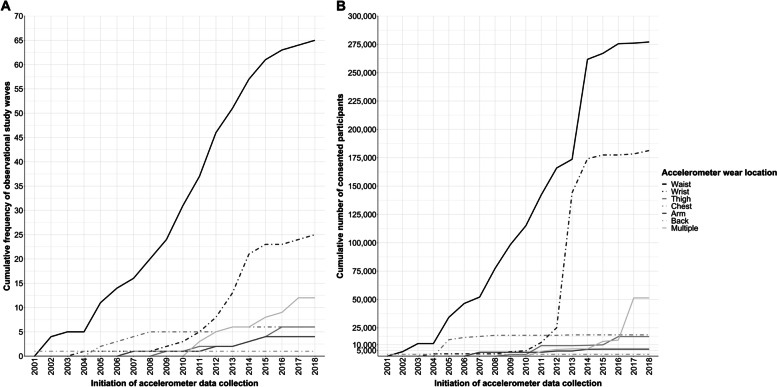
Cumulative frequency of observational studies of adult physical behaviours using accelerometers worn at different body locations (Panel A) and the cumulative number of participants (Panel B) Legend. Panel A shows the cumulative frequency of observational studies identified in this review that measure physical behaviours using accelerometers at different wear locations over time (from initiation of accelerometer measurement). Panel B shows the cumulative number of participants who consented to wear an accelerometer within these studies. Device wear locations are differentiated using different line styles

A total of 95 studies met the criteria for inclusion (Fig. 1). Of these, 15 included multiple accelerometer measures: either repeated cross-sectional measures of different samples of participants (e.g., National Health and Nutrition Examination Survey and Canadian Health Measures Survey), or repeated measures in the same sample (e.g. the SAGA Multi-Institutional Collaborative Cohort and Pelotas (Brazil) Birth Cohort studies). A total of 123 waves of accelerometer assessment were included in the review, these are described in full in Supplementary Table S2. Briefly, 69.1% (*n* = 85) of study waves were from prospective and 31.9% (*n* = 38) from cross-sectional studies. All included study waves originated in high (92.5%, *n* = 114) or upper middle income (8.3%, *n* = 9) countries (according to World Bank income classification [35]). Accelerometer measures were initiated between the year 2000 and 2018 (Fig. 2). Sample size ranged from 122 (Physical Activity through Sustainable Transport [PASTA]) to 106,053 (UK Biobank) participants (median 1412; Interquartile range 699—3421). Most commonly used devices were Actigraph (43.9%, *n* = 54), Actical (10.6%, *n* = 13), GENEactiv (7.3% *n* = 9), and activPal (5.6% *n* = 7). Devices were worn at the waist (52.8%, *n* = 65 study waves), wrist (20.3%, *n* = 25), chest (4.9%, *n* = 6), thigh (4.9%, *n* = 6), upper arm (3.3%, *n* = 4) and back (0.8%, *n* = 1). Sixteen study waves (13.0%) employed multiple devices, most often at two body locations. Of the 119 study waves for which distribution method determined, accelerometers were distributed to participants in person in 87.4% of study waves (*n* = 104), and via post for the remaining 12.6% (*n* = 15). Of the 113 study waves for which return method was determined, 37.2% (*n* = 42) collected devices from participants in person, 52.2% (*n* = 59) opted for device return via post, and 10.6% (*n* = 12) collected devices via another method (e.g., devices left at health care clinics or workplaces for collection by investigators). Of the 109 study waves that reported participant follow-up, participants were contacted during the measurement period in 15.6% of study waves (*n* = 17). The proportion of study-waves for which we were able to obtain data on each of the key study characteristics for this review is described in Table 2 and individual study data is described in Supplementary Table S2.

**Table 2:** Availability of data for review

Extracted information	N	(%)	Review Identification number
Study name	123	(100)	1–123
Study Country of origin	123	(100)	1–123
Study design (prospective or cross-sectional)	123	(100)	1–123
Year of start of physical activity measurement	123	(100)	1–123
Accelerometer make and model	123	(100)	1–123
Accelerometer body placement	123	(100)	1–123
Accelerometer wear instructions*	107	(86.9)	2–6, 8–28, 30–34, 37–47, 50–70, 72–77, 79–92, 97–114, 116–119, 121–123
Minimum accelerometer wear criteria*	103	(83.7)	1–26, 29–35, 38–47, 50- 60, 62–68, 71–80, 82–87, 90,91, 93–99, 104–107, 109–117, 119, 120, 121–123
Accelerometer distribution method	119	(96.7)	1–43, 45–47, 49–70, 72–114, 116–123
Accelerometer return method	113	(91.9)	1–32, 34–43, 45–47, 49–54, 56–59, 61 62, 64–70, 72–89, 91–114, 116–123
Participant follow up (yes or no)	109	(88.6)	1–4, 7–9, 11–25, 27–33, 35–47, 50–59, 61, 62, 64–70, 72–76, 78–89, 91–109, 111–114, 116–123
N invited to wear accelerometer	112	(91.1)	1–30, 33, 34, 36–43, 45–56, 58–65, 67, 68, 70–78, 80–98, 100–110, 112–117, 119–123
N consented to wear accelerometer	120	(97.6)	1–11, 13–109, 111–114, 116–123
Devices lost during measurement	88	(71.5)	1–9, 11–20, 24–29, 31–33, 35, 37–43, 45, 46, 50, 51, 53, 55, 57–59, 61, 62, 65–68, 71, 72, 78–80, 83 -85, 87, 92 -109, 111, 113, 114, 116–119, 121–123
N adhering to wear instructions	111	(90.2)	1–11, 13–26, 28, 29, 31–43, 45, 46, 47, 50–59, 61- 68, 70–77, 79 -102, 104 -111, 113 -117, 119 -123
Average wear time	86	(69.1)	1–3, 5- 9, 12–21, 23–28, 31–33, 35- 37, 39 -41, 45, 46, 50, 52, 55–59, 61, 62, 65–70, 72 -75, 78–87, 89, 90, 92, 97- 99, 104–108, 110, 111, 113 -117, 119, 121–123
Adverse events^†^	68	(55.3)	3–6, 8, 11, 13, 15–22, 24–28, 33, 36–40, 45, 47, 50–54, 56–59, 61, 62, 65, 67, 68, 70, 73, 74, 77–80, 82–89, 92, 97, 98, 104–107, 111, 113, 116, 122
Response bias	77	(62.6)	1–3, 5, 6, 8, 9, 11–14, 20 -24, 27, 28, 31, 33, 35–39, 41–43, 45–47, 51, 53–55, 59, 61, 62, 64, 67–69, 72–74, 77–83, 85–87, 93–96, 98, 100–102, 104–109, 111, 113, 117–122

Study waves and their review identification (ID) numbers are described in Supplementary Table S2. IDs listed for study waves for which data was identifiable to the review team and clear. Data was given the designation ‘unclear’ and excluded from the list above if it was not recorded, could not be identified from research articles or other publicly available documentation, was not obtained through correspondence with the study team, or otherwise could not be identified with certainty by the review team. *For reporting of accelerometer wear instructions and minimum wear criteria IDs listed denote studies for which the required number of wear days, and hours of wear per day was identifiable. †For reporting of adverse events, IDs listed denote studies for which recorded information regarding the number and/or nature of adverse events

### Meta-analyses of consent, data loss and adherence to wear criteria

#### Analyses of the proportion of invited participants who consented to wear an accelerometer

Data on both the number of people invited to wear an accelerometer and the number of people who consented was available for 110 study waves (89.4.0%). Two studies were excluded (IDs 54 and 92) as authors advised that the number who consented to wear a device reflected device availability rather than participant choice. Overall, the pooled estimate for the proportion of invited participants who consented to wear an accelerometer in the remaining 108 measurement waves was 75.0% (95%CI 70.0–80.0%). Between study variance was very large (I^2^ = 99.1%), ranging from 12 to 100%.

Study characteristics that were associated with consent to wear an accelerometer are described in Table 3. In-person distribution of accelerometers was associated with a higher rate of consent compared to postal distribution. Larger study sample size was also associated with higher consent rate and collectively these factors explained 27.3% of the between study variance in consent.

**Table 3:** Multivariate meta-regression analyses for proportion of consent, device loss and adherence to wear criteria

		**% Consented**				**% Loss**				**% Adhered**			
		b	(95% CI)	I^2^	Adj. R^2^	b	(95% CI)	I^2^	Adj. R^2^	b	(95% CI)	I^2^	Adj. R^2^
Accelerometer wear location	Waist	**Ref**		99.2%	27.3%	**Ref**		21.0%	70.1%	**Ref**		95.9%	12.3%
	Thigh	0.11	(-0.07, 0.30)			**0.04**	**(0.01, 0.08)**			0.05	(-0.11, 0.21)		
	Wrist	-0.02	(-0.09, 0.13)			-0.01	(-0.03, 0.01)			**0.14**	**(0.05, 0.23)**		
	Chest/Back/Arm	-0.07	(-0.22, 0.07)			0.01	(-0.01, 0.03)			0.10	(-0.01, 0.20)		
	Multiple	-0.13	(-0.29, 0.03)			**0.05**	**(0.02, 0.08)**			0.05	(-0.10, 0.20)		
Study design	Prospective	Ref				Ref				Ref			
	Cross-sectional	-0.01	(-0.10, 0.09)			-0.01	(-0.03, 0.01)			-0.03	(-0.10, 0.05)		
Sample size	Q1: 122–679	**Ref**				**Ref**				Ref			
	Q2: 680–1412	**0.09**	**(-0.04, 0.22)**			**0.01**	**(-0.02, 0.04)**			-0.08	(-0.18, 0.03)		
	Q3: 1412–3421	**0.06**	**(-0.06, 0.18)**			**0.03**	**(0.001, 0.06)**			-0.10	(-0.19, -0.01)		
	Q4: > 3422	**0.18**	**(0.03, 0.32)**			**0.02**	**(-0.01, 0.05)**			-0.07	(-0.19, 0.03)		
	*p *^*trend*^	**0.02**				**0.05**				0.28			
Distribution method	In person	**Ref**				Ref				**Ref**			
	Via post	**-0.30**	**(-0.42, -0.18)**			0.01	(-0.01, 0.03)			**-0.15**	**(-0.25, -0.04)**		
Return method	In person	Ref				Ref				Ref			
	Via post	-0.01	(-0.10, 0.12)			-0.01	(-0.1, 0.3)			0.07	(-0.02, 0.16)		
Follow-up	Yes	Ref				**Ref**				Ref			
	No	-0.08	(-0.20, 0.04)			**-0.04**	**(-0.05, -0.02)**			-0.01	(-0.11, 0.09)		
Study age	Q1: < 8yrs	Ref				Ref				Ref			
	Q2: 9-10yrs	0.08	(-0.04, 0.20)			0.001	(-0.02, 0.02)			-0.05	(-0.15, 0.05)		
	Q3: 11-14yrs	0.12	(-0.01, 0.23)			0.002	(-0.02, 0.02)			-0.02	(-0.11, 0.07)		
	Q4: > 14yrs	0.07	(-0.05, 0.20)			-0.01	(-0.03, 0.01)			-0.02	(-0.08, 0.12)		
	*p *^*trend*^	0.23				0.40				0.63			

Data are unstandardized beta (95%CI), therefore coefficients represent the difference in proportions of participants compared to the reference category. %consented = the proportion of participants invited to wear an accelerometer who consented to do so. %loss = the proportion of devices lost during data collection. %adhered = the proportion of consented participants who met minimum wear criteria for inclusion. I^2^ = between study variance, Adjusted R^2^ = the proportion of that between study variance explained by significant predictors within the model. Two studies (IDs #7 and #54) were excluded from analyses of %consented as the number consented was restricted by the device availability rather than only participant consent

### Analyses of the proportion of devices lost during accelerometer measurement

Data on both the number of people who consented to wear an accelerometer and the number of people for whom data was missing due to device loss during measurement was available in 69.9% (*n* = 86) of study waves. The pooled estimate for the proportion of data lost due to device loss was 1.0% (95%CI 0.05–1.3%). Between-study variance was large (I^2^ = 99.2%), ranging from 0 to 14%. In multivariate analyses (Table 3) higher loss was observed in study waves employing thigh-worn or multiple devices, compared to waist, and in larger studies, and those that contacted participants during measurement.

### Analyses of the proportion consented participants who adhered to minimum wear criteria

Data on the number of people who consented to wear an accelerometer and the number of people who adhered to minimum study wear criteria was available for 86.1% (*n* = 106) of study waves. Overall, the pooled estimate for the proportion of consented participants who adhered to minimum study wear criteria was 89.0% (CI 85.9–91.7%). Between study variance was very large (I^2^ = 99.7%), ranging from 40.3–100%.

Study characteristics associated with adherence rate in multivariate meta regression analyses were accelerometer wear location and accelerometer distribution method (see Table 3). Reported adherence tended to be higher in studies employing accelerometers worn at the wrist, relative to waist (reference category). In-person distribution of accelerometers was associated with higher rate of adherence relative to postal distribution. Collectively these factors explained 12.3% of between-study variance in adherence.

Sensitivity analyses following exclusion of repeated measures of the same participants from within prospective cohort studies or with multiple device measures did not markedly impact the results.

### Narrative syntheses

#### Accelerometer wear instructions, minimum wear criteria and average accelerometer wear

Supplementary Table S3 describes accelerometer wear instructions and minimum wear criteria according to accelerometer wear location. Overall participants were most commonly instructed to wear the accelerometer for seven consecutive days, except in study waves where accelerometers were worn at the chest, where monitoring periods tended to be shorter (66.7% ≤ 6 days). Wrist, thigh, chest, arm, and back accelerometers tended to be worn continuously (i.e., 24 h per day), whereas waist accelerometers tended to be worn during waking hours only (i.e., removed when sleeping). The most common minimum accelerometer wear-time criteria for waist and back accelerometers was ≥ 10 h of wear time per day for ≥ 4 days (occasionally including at least one weekend day). In contrast, study waves employing wrist, thigh, and chest accelerometers, required more hours of daily wear but fewer valid days. Where studies employed multiple devices, valid wear criteria were heterogenous, ranging from 4–16 h on 1–5 days.

**Fig. 3:**
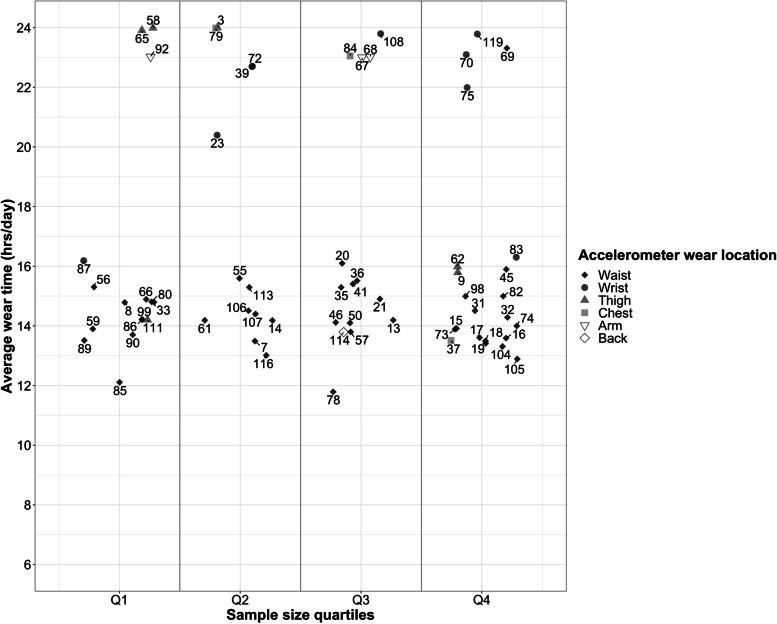
Average reported accelerometer wear (hrs/day) according to study sample size and accelerometer wear location Legend. Accelerometer wear (hours/day) according to study sample size (quartiles) and accelerometer wear location. Marker colour denotes accelerometer wear location. Study sample size quartiles were: Q1 = 122–629, Q2 = 630–1412, Q3 = 1412–3421, Q4 =  > 3421. Numeric values = study review ID number, connecting lines indicate studies with multiple measures (repeated cross-sectional measures of different samples, or repeated measures in the same sample)

Average daily accelerometer wear-time was determined in 69.9% of study waves (73.8% of waist; 44.0% of wrist; 100.0% of thigh; 50.0% of chest; 75.0% of arm; 100.0% of back, and 87.5% of those employing multiple devices). However, of study waves employing multiple devices, only 2 reported wear-time separately for different wear locations. For remaining study waves, this information was unavailable or unclear from available materials, was reported across the whole monitoring period rather than per day, or separately for different demographic groups. Figure 3 describes daily wear time according to wear location. Weartime tended to be highest for wrist and lower for waist accelerometers compared with other wear locations, irrespective of study design, sample size, distribution or return method, and participant follow-up.

### Response bias: Analyses of reported demographic differences between those invited and those who met wear-time criteria

Information regarding demographic differences between the invited and final study samples was determined for 62.6% of study waves (77 of 123 waves). Within these study waves the most common differences explored were age (48.1%, *n* = 37), sex (35.1%, *n* = 27), health status (31.2%, *n* = 24), socioeconomic status (SES; 29.9%, *n* = 23), body mass index (BMI; 26.0%, *n* = 20), ethnicity (10.4%, *n* = 8), and physical function (9.1%, *n* = 7). Across all device placements, participants in the final sample were more likely to be middle-aged, white, have a higher SES (typically classified by household income or educational attainment), have a lower BMI, and have better health and physical function. There was no clear evidence of sex differences between invited and final samples. In studies including adults of all ages, those in final samples were more likely to be older, and in studies of older adults were more likely to be younger.

### Adverse events: Reporting of adverse events attributable to accelerometer wear

Information on adverse events attributable to accelerometer wear were obtained for only 23.6% of all study waves (*n* = 29), However, of the 29 studies that reported information on adverse events, only 31.0% (*n* = 9) specified both the number of events and their nature (skin irritation/allergy [55.6%, *n* = 5]; discomfort [33.3%, *n* = 3]; and claustrophobia [11.1%, *n* = 1]), with the remaining 69.0% (*n* = 20) reporting general observations from the study team (e.g., “some instances of skin irritation”). Thirty-nine study waves (31.7%) reported no adverse events. In the remainder of study waves (44.7%, *n* = 55), it was unclear if a) adverse events were assessed and none were reported by participants or b) if adverse events were not assessed at all.

## Discussion

This review examined how methodological decisions regarding device-based physical behaviour measurement are associated with differences in participant recruitment, adherence to wear instructions and data loss. In doing so, we have identified several factors which may be important in the planning and execution of physical behaviour measurement in epidemiological studies. Average accelerometer wear-time tended to be lowest when devices were worn at the waist, and adherence to wear instructions highest in studies using wrist-worn devices. In person distribution of accelerometers was associated with both better rates of consent and better adherence to wear instructions. Based on our observations we make 3 principal recommendations for at-scale assessment of physical behaviours.

### Recommendation 1. Address inequalities in evidence

Almost all of the study waves included in the review originate in high income countries, reflecting previous observations that only a small percentage of physical behaviour research emanates from low- and middle-income-countries [36, 37]. Important socioeconomic and cultural differences, and competing healthcare priorities underpin the need for further population studies from low- and middle-income countries [38]. However, the resource intensive nature of accelerometer data collection, including costs of equipment, researcher time and expertise, and infrastructure for data management and analysis remains an important barrier [39]. Development of new measurement systems which make use of comparatively low-cost devices and widely available digital infrastructure overcome some of these barriers. Collaborative projects which facilitate the loaning of accelerometers, provision of detailed measurement protocols, resources and training for researchers and support for analyses can further facilitate expansion of global physical behaviour research [14, 40].

### Recommendation 2. Optimise data collection methods

Decisions regarding measurement of physical behaviours may be made for myriad scientific and practical reasons. Based on observations from the current review, we recommend careful consideration of accelerometer wear location, distribution method and response bias.

i. Accelerometer wear location.
  Decisions on accelerometer wear location are often complex. Researchers must balance competing factors including the study population, cost of devices and their deployment, participant burden associated with different wear protocols, and the behavioural constructs of interest (e.g., posture). [41–43] Our findings suggest that accelerometer wear location may also be associated with data collection outcomes. Wear-time tended to be lower when using waist-worn accelerometers, while adherence with minimum wear criteria was highest when accelerometers were worn at the wrist. This may reflect differences in instructions for participants, as many studies employing waist worn devices required wear only during waking hours. However, findings are consistent with previous research reporting that simple ‘wrist-watch-like’ attachment of accelerometers is less burdensome for participants [12] and associated with higher wear compliance [16, 24]. Developments in data processing and analytical approaches continue to shift the landscape regarding which behavioural constructs can be captured by accelerometers at different locations. However behavioural assessment for complete 24 h periods may be best achieved using devices which do not require removal (e.g., waterproof devices worn at the thigh), and wear-time may prove highest when accelerometers are worn at the wrist [19].
ii. Accelerometer distribution method.
  In-person distribution of devices was associated with a substantially higher proportion of participants both consenting to wear an accelerometer and meeting minimum wear criteria compared to distribution by post. In-person distribution, or ‘fitting’ of accelerometers by investigators, can ensure correct and comfortable attachment, development of rapport between participants and investigators, and allows participants’ questions about the accelerometer’s care and function during the measurement period to be addressed. For studies that are smaller in terms of sample size or geographical coverage, in-person distribution may be possible, while for larger studies postal distribution of devices may be necessitated. Nevertheless, where in-person distribution is feasible, the potential additional cost should be weighed against possible improvements in recruitment and adherence, estimates of physical behaviours and generalisability of findings.
iii. Response bias.
  This review supports previous observations that participants who wear accelerometers tend to be middle-aged, white, healthier and from a higher socioeconomic position, [44, 45]. This mirrors the response bias commonly observed with other measures, and may compound the healthy volunteer effect observed in recruitment for observational studies. This demographic bias has important implications for the design and undertaking of physical behaviour research, particularly if inferences are to be made about the prevalence/distribution of these behaviours to a wider population. Over-sampling of individuals with demographic characteristics associated with poorer recruitment rates (identified here and previously), or use of incentives, have potential to reduce bias in recruitment. However, exclusion of participants without high levels of wear time (e.g., 24 h on multiple days) may introduce further bias due to demographic differences in wear-time [46].

### Recommendation 3. Standardise reporting for comparison and harmonisation

The next generation of population physical behaviour research is likely to involve global expansion of cohort and surveillance studies, [47] and consortia like ProPASS [14], https://www.ispah.org/ispah-propass/) that bring together individual participant data with the statistical power to provide detailed understanding of relationships between complex multidimensional behaviours and lifelong health [14]. Key to this expansion is the sharing of optimal and developing practice for data collection, and the comprehensive reporting of data collection methods and outcomes, which can facilitate between-study comparison, and prospective and retrospective harmonisation of data. Storage of raw unprocessed acceleration data from accelerometers allows for common analysis methods (e.g., determination of wear time and behavioural metrics) to be applied across studies, and for the application of new analytical methods as they continue to develop at pace. Consistency in reporting of methods in accelerometer studies has improved, particularly in response to the publication of influential papers [42, 43]. However, while much of the information sought for this review was available from research articles, publicly available documentation, and via support from study teams (for which we are extremely grateful), significant gaps remained. In the interests of supporting the expansion of population physical activity research [42] In Supplementary Figure S4 we provide a reporting checklist as a practical resource for researchers to support this.

### Strengths and limitations

To our knowledge, this is the first review to identify methodological factors which contribute to successful collection of data on physical behaviours in observational studies. Informed by our findings, we provide recommendations for the conduct of large-scale physical behaviour measurement, which may benefit the development of new cohort studies and expansion of consortia. This review was conducted and reported in accordance with best practice guidance, although it is not without limitation. While we aimed to identify all studies waves meeting our inclusion criteria, it is possible that some eligible studies were not identified despite the rigorous multi-stage process followed. The information synthesised in this review was extracted from published research articles, online study materials and correspondence with study investigators. Nevertheless, responses were not received from all study teams that were contacted for information prior to publication. Data regarding average accelerometer wear was on occasion calculated by study teams following exclusion of participants with very low wear, which may have inflated some reported average wear time values. Our aim in these analyses was to provide a high-level appraisal of associations between study characteristics and data collection outcomes. In doing so we interpret these findings conservatively in the knowledge that cohort studies vary considerably in ways not captured in this review and as such there is a possibility of confounding.

## Conclusion

In summary, population assessment of free-living physical behaviours using accelerometers worn at various body locations will increasingly play a key role in our understanding of links between physical behaviours and lifelong health. Methodological decisions will influence important study outcomes including participant recruitment and compliance. Continuous 24 h assessment may be best achieved using devices worn at the wrist or thigh. In-person distribution of devices may benefit participant recruitment and adherence to wear instructions. More studies are needed from low- and middle-income countries. Comprehensive reporting of accelerometer data collection methods and outcomes can support the development of new future studies and international consortia. These offer exciting potential for unprecedented understanding of behaviour-health relationships, and for informing international research priorities, policy and public health practice.

## Supplementary Information

**Additional file 1.** Supplementary figure 1. Example search strategy

**Additional file 2.** Supplementary table S2. Extracted data for each study wave

**Additional file** **3. **Supplementary table S2 legend. Supplementary Table 1. Extracted data for each study wave included in this review.Start of data collection: refers to the initiation of accelerometer data collection. N Invited: the number of participants invited to wear an accelerometer. N Consented: the number of participants who consented to wear an accelerometer. N Lost: the number of devices lost during measurement. N Adhered: the number of participants who met the minimum accelerometer wear criteria. Information sources: i. References: The primary reference for information regarding a study wave. ii. Data provided or verified by study team: ‘Yes’ indicates that a member of the study team (principal investigator, co-investigator, author) provided some or all of the data described, and/or verified the accuracy of the extracted data. ‘Unclear: the designation unclear indicates that the information was not available from published articles, publicly available documentation or through correspondence with the study team, was not measured or recorded, or otherwise could not be confirmed with certainty by the review team.  

## Data Availability

The data supporting the conclusions of this article are included within the article and its additional files.

## Abbreviations

PA: Physical Activity
SB: Sedentary Behaviour
PRISMA: Preferred Reporting Items for Systematic Reviews and Meta-Analyses
SWiM: Systematic Review Without Meta-Analysis
