# Supplementary material for: The impact of selected methodological factors on data collection outcomes in observational studies of device-measured physical behaviour in adults: A systematic review
Source: Int J Behav Nutr Phys Act. 2023 Mar 8;20:26. doi: 10.1186/s12966-022-01388-9 (PMC9993720; doi:10.1186/s12966-022-01388-9)
Supplement: Supplementary file 1 — Additional file 1. Supplementary figure 1. Example search strategy [file 12966_2022_1388_MOESM1_ESM.pdf]

Supplementary figure 1. Example search strategy

Terms

1. (physical adj2 (activit* or inactivit* or behavio* or exercise).ti.ab

2. (sedentary adj2 (activit* or behavio*). ti.ab

3. (sitting adj2 (time or behavio*). ti.ab

4. accleromet* ti.ab

5. (activity adj2 (monitor* or device). ti.ab

6. motion sensor. ti.ab

7. inclinometer. ti.ab

8. observational adj2 (stud* or cohort). ti.ab

9. cohort adj2 (stud*). ti.ab

10. cross-sectional. ti.ab

11. prospective. ti.ab

Example strategy

1 or 2 or 3

and

4 or 5 or 6 or 7

and

8 or 9 or 10 or 11

Search Limits:

Participants: adult, human

Articles: Journal articles, English, Full-text, Journal articles, published in English, full text available*
