# Supplementary material for: The impact of selected methodological factors on data collection outcomes in observational studies of device-measured physical behaviour in adults: A systematic review
Source: Int J Behav Nutr Phys Act. 2023 Mar 8;20:26. doi: 10.1186/s12966-022-01388-9 (PMC9993720; doi:10.1186/s12966-022-01388-9)
Supplement: Supplementary file 2 — Additional file 2. Supplementary table S2. Extracted data for each study wave [file 12966_2022_1388_MOESM2_ESM.docx]

|  | | | | | | | | | | | | | | | | | | Information sources | |
| --- | --- | --- | --- | --- | --- | --- | --- | --- | --- | --- | --- | --- | --- | --- | --- | --- | --- | --- | --- |
| Review ID | Study Name | Study Design | Start of data collection | Wear Location | Accelerometer make and model | Accelerometer wear instructions | Dist. Method | Return Method | Participant Follow-up? | N Invited | N Consented | N Devices Lost | Minimum wear criteria | N Adhered | Ave. wear-time | Response bias: accelerometer wearers were: (↑) more likely to be, (↔) no difference | Adverse events | References (journal articles or published reports) | Data provided or verified by study team |
| 1 | ACT: Adult Changes in Thought Study | Prospective | 2016 | Multiple (thigh) | ActivPAL micro (PAL Technologies Ltd., Glasgow, UK) | Continuous, no. days unclear | In-person | Post | No | 1757 | 1135 | 56 | 10 hrs, on 4 days | 1039 | 15.4 hrs.d | ↑ younger, better health | Not systematically recorded | .(1) | Yes |
| 2 | ACT: Adult Changes in Thought Study | Prospective | 2016 | Multiple (waist) | Actigraph wGT3X (Actigraph, Pensacola, Florida, USA) | Continuous, no. days unclear, removal for water activities | In-person | Post | No | 1757 | 1151 | 35 | 10 hrs, on 4 days | 1088 | 15.1 hrs.d | ↑ younger, better health | Not systematically recorded | .(1) | Yes |
| 3 | ActiFE Ulm study: Activity and Function in the Elderly in Ulm | Prospective | 2009 | Thigh | ActivPAL (PAL Technologies Ltd., Glasgow, UK) | Continuous, 7 days | In-person | In-person | No | 1506 | 1333 | 0 | 24 hrs, on 2 days | 1271 | 24 hrs.d | ↑ better health | 0 recorded | .(2) | Yes |
| 4 | AGES II:Age Gene/Environment Suscetability Study II | Prospective | 2009 | Waist | ActiGraph GT3X (Actigraph, Pensacola, Florida, USA) | Waking hrs, 7 days, removal for water activities | In-person | In-person | No | 1194 | 671 | 5 | 10 hrs, on 4 days | 579 | Unclear | Unclear | 0 recorded | .(3-5) |  |
| 5 | AGNES | Cross-sectional | 2017 | Multiple (thigh) | UKK RM42 (UKK Terveyspalvelut Oy, Tampere, Finland) + eMotion Faros 180 (BlindSight, Frondenberg, Germany) | Continuous, 7 days | In-person | In-person | Unclear | 910 | 495 | 2 | 24 hrs, on 3 days | 486 | 24 hrs.d | ↑ higher physical activity, co-habiting, poorer physical function | Yes, specific (1, discomfort) | .(6) | Yes |
| 6 | AGNES | Cross-sectional | 2017 | Multiple (chest) | UKK RM42 (UKK Terveyspalvelut Oy, Tampere, Finland) + eMotion Faros 180 (BlindSight, Frondenberg, Germany) | Continuous, 8 days | In-person | In-person | Unclear | 910 | 464 | 0 | 24 hrs, on 3 days | 441 | 24 hrs.d | ↑ higher physical activity, co-habiting, poorer physical function | Yes, specific (1, discomfort) | .(6) | Yes |
| 7 | ALECS HK: Active Lifestyle and the Environment in Chinese Seniors | Cross-sectional | 2012 | Waist | Actigraph GT3X (Actigraph, Pensacola, Florida, USA) | Waking hrs, no. days unclear, removal for water activities | In-person | In-person | No | 416 | 416 | 0 | 10 hrs, on 5 days | 11 | 13.5 hrs.d | Unclear | Not systematically recorded | .(7) |  |
| 8 | AusDiab: The Australian Diabetes Obesity and Lifestyle Study | Prospective | 2005 | Waist | Actigraph WAM 7164 (Actigraph, Pensacola, Florida, USA) | Waking hrs, 7 days | In-person | In-person | Yes | 202 | 148 | 0 | 10 hrs, on 5 days | 143 | 14.8 hrs.d | ↑ higher SES (income & education); ↔ BMI | 0 recorded | .(8, 9) | Yes |
| 9 | BCS70: 1970 British Birth Cohort Study | Prospective | 2016 | Thigh | ActivPal3 micro (PAL Technologies Ltd., Glasgow, UK) | Continuous, 7 days | In-person | Post | No | 7439 | 6562 | 591 | 24 hrs, on 1 day | 5448 | 15.8 hrs.d | ↑ female, non-smoker, better health, lower BMI | Not systematically recorded | .(10, 11) | Yes |
| 10 | BEPAS: Belgian Environmental PA Study | Cross-sectional | 2010 | Waist | Actigraph GT3X (Actigraph, Pensacola, Florida, USA) | Continuous, 7 days | In-person | In-person | Unclear | 1135 | 508 | Unclear | 10 hrs, on 5 days | 438 | Unclear | Unclear | Not systematically recorded | .(12) | Yes |
| 11 | BLSA: Baltimore Longitudinal Study of Aging | Prospective | 2007 | Chest | Actiheart combined heart rate and activity monitor | Continuous, 7 days | In-person | Post | No | 1330 | 888 | 2 | 24 hrs, on 3 days | 849 | Unclear | ↑ younger, better health | Yes, generic (some instances of skin irritation) | .(13) | Yes |
| 12 | BLSA: Baltimore Longitudinal Study of Aging | Prospective | 2015 | Wrist | Actigraph GT9X (Actigraph, Pensacola, Florida, USA) | Continuous, 7 days | In-person | Post | No | 1330 | Unclear | 1 | 21.6 hrs, on 3 days | Unclear | 23.96 hrs.d | No differences | Not systematically reported | .(14) | Yes |
| 13 | British Regional Heart Study | Prospective | 2010 | Waist | Actigraph GT3X (Actigraph, Pensacola, Florida, USA) | Waking hrs, 7 days, removal for water activities | Post | Post | No | 3292 | 1848 | 19 | 10 hrs, on 3 days | 1644 | 14.2 hrs.d | ↑ younger, lower BMI | 0 recorded | .(15, 16) | Yes |
| 14 | British Women’s Heart and Health Study | Prospective | 2010 | Waist | Actigraph GT3x (Actigraph, Pensacola, Florida, USA) | Waking hrs, 7 days, removal for water activities | Post | Post | No | 3237 | 1365 | 101 | 10 hrs, on 3 days | 946 | 14.2 hrs.d | ↑ lower BMI | Not systematically recorded | .(15, 17) | Yes |
| 15 | Canadian Health Measures Survey, cycle 1 (2007-2009) | Cross-sectional | 2007 | Waist | Actical accelerometer (Phillips – Respironics, Oregon, USA) | Waking hrs, 7 days | In-person | Post | Yes | 5594 | 5412 | 128 | 10 hrs, on 4 days | 4441 | 13.9 hrs.d | Unclear | 0 recorded | .(18) | Yes |
| 16 | Canadian Health Measures Survey, cycle 2 (2009-2011) | Cross-sectional | 2009 | Waist | Actical accelerometer (Phillips – Respironics, Oregon, USA) | Waking hrs, 7 days | In-person | Post | Yes | 6393 | 6331 | 130 | 10 hrs, on 4 days | 5660 | 13.6 hrs.d | Unclear | 0 recorded | .(19) | Yes |
| 17 | Canadian Health Measures Survey, cycle 3 (2012-2013) | Cross-sectional | 2012 | Waist | Actical accelerometer (Phillips – Respironics, Oregon, USA) | Waking hrs, 7 days | In-person | Post | Yes | 5783 | 5545 | 458 | 10 hrs, on 4 days | 4917 | 13.6 hrs.d | Unclear | 0 recorded | .(19) | Yes |
| 18 | Canadian Health Measures Survey, cycle 4 (2014-2015) | Cross-sectional | 2014 | Waist | Actical accelerometer (Phillips – Respironics, Oregon, USA) | Waking hrs, 7 days | In-person | Post | Yes | 5791 | 5635 | 264 | 10 hrs, on 4 days | 4853 | 13.5 hrs.d | Unclear | 0 recorded | .(19) | Yes |
| 19 | Canadian Health Measures Survey, cycle 5 (2016-2017) | Cross-sectional | 2016 | Waist | Actical accelerometer (Phillips – Respironics, Oregon, USA) | Waking hrs, 7 days | In-person | Post | Yes | 5780 | 5573 | 230 | 10 hrs, on 4 days | 4880 | 13.4 hrs.d | Unclear | 0 recorded | .(19) | Yes |
| 20 | Cancer prevention study 3 [[CPS-3 Activity Validation Sub-study]] | Cross-sectional | 2015 | Waist | Actigraph GT3x  (Actigraph, Pensacola, Florida, USA) | Waking hrs, 7 days, removal for water activities | Post | Post | No | 10000 | 1801 | 0 | 10 hrs, on 4 days | 726 | 16.1 hrs.d | ↑ female, older | Yes, specific (1, claustrophobic) | .(20) | Yes |
| 21 | CARDIA: Coronary Artery Risk Development in Young Adults Study | Prospective | 2005 | Waist | Actigraph 7164 and wGT3X-BT (Actigraph, Pensacola, Florida, USA) | Waking hrs, 7 days, removal for water activities | In-person | Post | Yes | 3549 | 3001 | Unclear | 10 hrs, on 4 days | 2080 | 14.9 hrs.d | ↑ female, older, white, higher SES (income & education), lower BMI, better health | 0 recorded | .(21) | Yes |
| 22 | CoLAUS Study | Prospective | 2014 | Wrist | GENEActiv (Active Insights, Kimbolton, Cambridgshire, UK) | Continuous, 14 days | In-person | Post | No | 4881 | 3060 | Unclear | 10 hrs (wkday) and 8 hrs (wkend) , 5 wkdays and 2 wkend days | 2944 | Unclear | ↑ younger, male, higher SES (occupation), better health | 0 recorded | .(22, 23) | Yes |
| 23 | COMO VAI? | Prospective | 2014 | Wrist | GENEActiv (Active Insights, Kimbolton, Cambridgshire, UK) | Continuous, 7 days | In-person | Other | No | 1451 | 973 | Unclear | 16 hrs, on 2 days | 973 | 20.4 hrs.d | ↑ SES | Not systematically recorded | .(24) |  |
| 24 | Copenhagen City Heart Study | Prospective | 2011 | Multiple(thigh, waist) | Actigraph GT3X+ (Actigraph, Pensacola, Florida, USA) | Continuous, 7 days | In-person | In-person | No | 4543 | 2335 | 316 | 16 hrs, on 5 days | 1670 | 23.8 hrs.d | ↑ younger, lower BMI, higher SES, better health | Yes, generic (some instances of skin irritation and soreness) | .(25) | Yes |
| 25 | Danish National Survey of Diet and Physical Activity (2007) | Cross-sectional | 2007 | Multiple(waist, knee) | Yamax SW 200 (Bridgnorth, Shropshire, UK) | Waking hrs, 7 days | In-person | Other | Yes | 368 | 224 | 0 | 10 hrs, on 4 days | 203 | 15.1 hrs.d | Unclear | Yes, generic (some instances of skin irritation) | .(26) |  |
| 26 | Danish National Survey of Diet and Physical Activity (2011) | Cross-sectional | 2011 | Multiple (waist, knee) | Yamax SW 200 (Bridgnorth, Shropshire, UK) | Waking hrs, 7 days | In-person | Other | Unclear | 2925 | 1515 | 0 | 10 hrs, on 4 days | 1157 | 14.8 hrs.d | Unclear | Yes, generic (some instances of skin irritation) | .(26) |  |
| 27 | DOSES: Danish Observational Study of Eldercare work and musculoskeletal disorders | Prospective | 2013 | Multiple(thigh, back, arm) | Actigraph GT3X+ (Actigraph, Pensacola, Florida, USA) | Continuous, 4 days, removal for water activities | In-person | Other | Yes | 941 | 470 | 18 | Unclear (4 hrs, or 75% of shift) | Unclear | 13.1 hrs.d | ↑ poorer health (neck/shoulder pain) | Yes, generic (some instances of skin irritation) | .(27) | Yes |
| 28 | DPHACTO: The Danish Physical activity cohort with objective measurements | Prospective | 2012 | Multiple (thigh, waist, arm, back) | Actigraph GT3X + Actigraph (Actigraph, Pensacola, Florida, USA) | Continuous, 4-6 days | In-person | Other | No | 1087 | 909 | 0 | 10 hrs, required no. days unclear | 839 | 15.9 hrs.d | ↑ better health, lower alcohol consumption | Yes, generic (some instances of skin irritation) | .(28) | Yes |
| 29 | ECHO-SOL: Echocardiographic Study of Latinos | Cross-sectional | 2011 | Waist | Actical (Phillips – Respironics, Oregon, USA) | Waking hrs, no. days unclear, removal for water activities | In-person | In-person | No | 1818 | 1818 | 0 | 10 hrs, on 3 days | 1353 | Unclear | Unclear | Not systematically recorded | .(29) |  |
| 30 | EGIR-RISC Study: European Group for the study of Insulin Resistance: Relationship between IS and CVD | Prospective | 2002 | Waist | Actigraph AM7164-2.2 (Actigraph, Pensacola, Florida, USA) | Waking hrs, 8 days, removal for water activities | In-person | In-person | No | 1259 | 847 | Unclear | 10 hrs, on 3 days | Unclear | Unclear | Unclear | Not systematically recorded | .(30) | Yes |
| 31 | EPIC Norfolk (wave 1, health check 3): European Prospective Investigation into Cancer | Prospective | 2006 | Waist | ActiGraph GT1M (Actigraph, Pensacola, Florida, USA) | Waking hrs, 7 days, removal for water activities | In-person | Post | No | Unclear | 4148 | 52 | 10 hrs, on 4 days | 4052 | 14.5 hrs.d | ↔ sex, age, SES (education), BMI, health | Not systematically recorded | .(31, 32) | Yes |
| 32 | EPIC Norfolk (waves 1 and 2, health checks 3 and 4): European Prospective Investigation into Cancer | Prospective | 2006 | Waist | ActiGraph GT1M/GT3X+ (Actigraph, Pensacola, Florida, USA) | Waking hrs, 7 days | In-person | Post | No | Unclear | 7820 | 80 | 10 hrs, on 4 days | 7686 | 14.3 hrs.d | Unclear | Not systematically recorded | .(33, 34) | Yes |
| 33 | EpiFloripa Aging study | Prospective | 2013 | Waist | Actigraph GT3X and GT3X + (Actigraph, Pensacola, Florida, USA) | Waking hrs, no. days unclear, removal for water activities | In-person | Unclear | No | 533 | 484 | 0 | 10 hrs, on 4 days | 425 | 14.8 hrs.d | ↑ better health | 0 recorded | .(35) |  |
| 34 | EPIMOV: Epidemiology and Human Movement Study | Prospective | 2013 | Waist | Actigraph GT3x + (Actigraph, Pensacola, Florida, USA) | Waking hrs, 7 days, removal for water activities | In-person | In-person | Unclear | 1257 | 1040 | Unclear | 10 hrs, on 4 days | 1040 | Unclear | Unclear | Not systematically recorded | .(36) |  |
| 35 | Estudio Latinoamericano de Nutricion y Salud adult study/Latin American Study of Nutrition and Health | Cross-sectional | 2014 | Waist | Actigraph GT3X+ (Actigraph, Pensacola, Florida, USA) | Waking hrs, no. days unclear, removal for water activities | In-person | In-person | No | Unclear | 3687 | 0 | 10 hrs, on 7 days | 2524 | 15.3 hrs.d | ↑ urban area | Not systematically recorded | .(37) |  |
| 36 | EVIDENT | Prospective | 2010 | Waist | Actigraph GT3X (Actigraph, Pensacola, Florida, USA) | 7 days, wear instructions unclear | In-person | In-person | No | 2696 | 1553 | Unclear | Required no. hrs.d unclear, on 4 days | 1300 | 15.5 hrs.d | ↔ sex, age | 0 recorded | .(38) |  |
| 37 | Fenland Study | Prospective | 2005 | Chest | Combined heart rate and uniaxial movement sensor (Actiheart, CamNtech, Papworth, UK) | Continuous, 6 days | In-person | Post | No | 46024 | 12435 | 25 | Unclear (≥ 72 hrs, or ≥ 8 hrs through each quadrant of the day) | 12002 | 13.5 hrs.d | No differences | Yes, specific (40, skin irritation) | .(39) | Yes |
| 38 | FINRISK | Cross-sectional | 2012 | Waist | Hookie accelerometer (Traxmeet Ltd., Espoo, Finland) | Waking hrs, 7 days, removal for water activities | In-person | Post | No | 3268 | 1204 | 0 | 10 hrs, on 4 days | 1122 | Unclear | ↑ older | 0 recorded | .(40) |  |
| 39 | FIREA (2014): Finnish Retirement and Aging study | Prospective | 2014 | Wrist | ActiGraph wActiSleep-BT (ActiGraph, Pensacola, Florida, USA) | Continuous, 7 days | Post | Post | No | 2663 | 908 | 1 | 10 hrs, on 4 days | 873 | 22.7 hrs.d | ↑ female, higher SES (occupation), higher physical activity | Yes generic (very few with skin irritation) | .(41) | Yes |
| 40 | FIREA (2015): Finnish Retirement and Aging study | Prospective | 2015 | Multiple (thigh, wrist) | ActiGraph wActiSleep-BT (ActiGraph, Pensacola, Florida, USA) | Continuous, 4 days, removal for water activities | Post | Post | No | 773 | 290 | 0 | 10 hrs, on 1 days | 287 | 15.7 hrs.d | Unclear | Yes, generic (some instances of skin irritation) | .(42, 43) | Yes |
| 41 | Framingham Heart Study. Generation 3 cycle 2 | Prospective | 2008 | Waist | Actical (Phillips – Respironics, Oregon, USA) | Continuous, 8 days | In-person | Post | No | 3800 | 3421 | 118 | 10 hrs, on 3 days | 2944 | 15.4 hrs.d | ↑ lower BMI | Not systematically recorded | .(44) | Yes |
| 42 | Framingham Generation 3 cycle 3 - Generation 3 follow-up | Prospective | 2016 | Waist | Actical (Phillips – Respironics, Oregon, USA) | Waking hrs, 8 days | In-person | Post | No | 3521 | 2898 | 91 | 10 hrs, on 3 days | 2426 | Unclear | ↑ lower BMI | Not systematically recorded |  | Yes |
| 43 | Framingham Generation 2 cycle 9 | Prospective | 2011 | Waist | Actical (Phillips – Respironics, Oregon, USA) | Waking hrs, 8 days | In-person | Post | No | 2713 | 1966 | 26 | 10 hrs, on 3 days | 1765 | Unclear | ↑ lower BMI | Not systematically recorded |  | Yes |
| 44 | HANDLS study: Healthy Aging in Neighbouhoods of Diversity Across the Lifespan | Prospective | 2013 | Wrist | ActiGraph GT3X+ (Actigraph, Pensacola, Florida, USA) | Continuous, 7 days | Unclear | Unclear | Yes | Unclear | 760 | Unclear | 10 hrs, on 4 days | Unclear | Unclear | Unclear | Not systematically recorded | .(45) | Yes |
| 45 | HCHs/SOL: Hispanic Community Health Study/Study of Latinos. Sueno Ancillary Study | Prospective | 2008 | Waist | Actical (Phillips – Respironics, Oregon, USA) | Waking hrs, 7 days, removal for water activities | In-person | Post | Yes | 16415 | 16415 | 1262 | 10 hrs, on 3 days | 12750 | 15.9 hrs.d | ↑ male, older, higher SES (income), lower BMI; ↔ SES (education), health | 0 recorded | .(46) | Yes |
| 46 | Health 2011 | Cross-sectional | 2011 | Waist | Hookie AM 20, Traxmeet Ltd, Espoo, Finland | Waking hrs, 7 days, removal for water activities | In-person | Post | No | 2455 | 2040 | 15 | 10 hrs, on 4 days | 1587 | 14.1 hrs.d | ↑ higher SES (education), better health | Not systematically recorded | .(47, 48) | Yes |
| 47 | Health Survey for England | Cross-sectional | 2008 | Waist | Actigraph GT1M (Actigraph, Pensacola, Florida, USA) | Waking hrs, 7 days | In-person | In-person | No | 4507 | 3380 | Unclear | 10 hrs, on 4 days | 2115 | Unclear | ↑ middle-aged | 0 recorded | .(49) | Yes |
| 48 | Healthy Aging Initiative (Umea) | Prospective | 2012 | Waist | Actigraph GT3X+ (Actigraph, Pensacola, Florida, USA) | 7 days, wear instructions unclear | Unclear | Unclear | Unclear | 1390 | 1390 | Unclear | Unclear | Unclear | Unclear | Unclear | Not systematically recorded | .(50) |  |
| 49 | Helsinki Birth Cohort | Prospective | 2012 | Arm | SenseWear Pro 3 (BodyMedia, Pittsburgh, USA) | 10 days, wear instructions unclear | In-person | In-person | Unclear | 1404 | 1094 | Unclear | Required no. hrs.d unclear, on 5 days | Unclear | Unclear | Unclear | Not systematically recorded | .(51) |  |
| 50 | Hisyama | Prospective | 2009 | Waist | Active style Pro HJA-350IT (Omron Healthcare, Kyoto, Japan) | Continuous, 7 days, removal for water activities | In-person | In-person | No | 2247 | 1987 | 3 | 10 hrs, on 4 days | 1740 | 14.1 hrs.d | Unclear | 0 recorded | .(52) |  |
| 51 | HoTN: The Health of the Nation survey | Cross-sectional | 2013 | Chest | Actiheart (Camtech Ltd, Fenstanton, Cambridge, UK) | Continuous, 7 days | In-person | In-person | No | 527 | 364 | 0 | 24 hrs, on 1 day | 354 | Unclear | No differences | Yes, generic (some instances of skin irritation) | .(53) | Yes |
| 52 | The HUNT Study (Trondelag Health Study) | Prospective | 2017 | Multiple (thigh, back) | Axivity AX3 (Axivity Ltd, Newcastle Upon Tyne, UK) | Continuous, 7 days | In-person | Post | No | 55561 | 32794 | Unclear | 24 hrs on 3 days | 26127 | 24hrs.d | Unclear | Yes, generic (some instances of skin irritation) |  | Yes |
| 53 | Inuit Health in Transition Study, Greenland | Cross-sectional | 2005 | Chest | Actiheart (Camtech Ltd, Fenstanton, Cambridge, UK) | Continuous, 5 days | In-person | In-person | No | 3102 | 2055 | 0 | 24 hrs, on 2 days | 1995 | Unclear | ↑ younger | Yes, generic (some instances of skin irritation) | .(54) |  |
| 54 | The Irish Longitudinal Study of Aging | Prospective | 2014 | Wrist | GENEActiv (Active Insights, Kimbolton, Cambridgshire, UK) | Continuous, 7 days | In-person | Post | No | 5317 | 1578 | Unclear | 24 hrs, on 4 days | 1533 | Unclear | ↑ retired | Yes, specific (2, 1 irritation and 1 rash) | .(55) | Yes |
| 55 | Iwaki Health Promotion Project | Cross-sectional | 2018 | Waist | HW-100 (Kao Corporation, Tokyo, Japan), | Waking hrs, 10 days, removal for water activities | In-person | Unclear | No | 10000 | 1056 | 0 | 10 hrs, on 7 days | 758 | 15.6 hrs.d | ↑ female, older | Not systematically recorded | .(56) |  |
| 56 | Kora: Kooperative Gesundheitsforschung in der Region Augsburg | Prospective | 2013 | Waist | Actigraph GT3X+ (Actigraph, Pensacola, Florida, USA) | Waking hrs, 7 days | In-person | Post | No | 1043 | 589 | Unclear | 10 hrs, on 4 days | 477 | 15.3 hrs.d | Unclear | Yes, generic (only a few, annoying) | .(57, 58) | Yes |
| 57 | Korea National Health and Nutrition Examination Survey 2014-2015 | Cross-sectional | 2014 | Waist | Actigraph GT3X+ (Actigraph, Pensacola, Florida, USA) | Waking hrs, 7 days, removal for water activities | In-person | In-person | No | Unclear | 1827 | 9 | 10 hrs, on 1 day | 1704 | 13.8 hrs.d | Unclear | 0 recorded | .(59) | Yes |
| 58 | LBC1936: The Lothian Birth Cohort 1936 | Prospective | 2014 | Thigh | activPAL monitor (activPAL3c, PAL Technologies Ltd, Glasgow, UK) | Continuous, 7 days | In-person | In-person | No | 374 | 304 | 2 | 24 hrs, on 7 days | 283 | 24 hrs.d | Unclear | Yes, specific (8, skin irritation) | .(60) |  |
| 59 | LISPE: Life-Space Mobility in Older people | Prospective | 2012 | Waist | Hookie, tri-axial, “AM20 Activity Meter”, Hookie Technologies Ltd, Espoo, Finland | Waking hrs, 7 days, removal for water activities | In-person | In-person | No | 461 | 190 | 1 | 10 hrs, on 4 days | 175 | 13.9 hrs.d | ↑ younger, better physical function; ↔ sex, health | 0 recorded | .(61-63) |  |
| 60 | LOFUS: Lolland-Falster Denamrk | Cross-sectional | 2017 | Multiple (thigh, back) | Axivity AX3 (Axivity LtD, Newcastle Upn Tyne, UK) | Continuous, 7 days | In-person | Unclear | Unclear | 17952 | 3904 | Unclear | 8 hrs, on 4 days | Unclear | Unclear | Unclear | Not systematically recorded | .(64) |  |
| 61 | Longitudinal Aging Study. Amsterdam | Prospective | 2015 | Waist | ActiGraph GT3X (Actigraph, Pensacola, Florida, USA) | Waking hours, 7 days, removal for water activities | Post | Post | Yes | 1770 | 1412 | 24 | Required no. hrs.d unclear, on 4 days | 1218 | 14.2 hrs.d | ↑ male, younger, lower BMI | 0 recorded | .(65) | Yes |
| 62 | The Maastricht study | Prospective | 2011 | Thigh | ActivPal3 (PAL Technologies Ltd., Glasgow, UK) | Continuous, 8 days | In-person | In-Person | No | 8838 | 7921 | 32 | 10 hrs, on 1 day | 7606 | 16 hrs.d | ↑ older, ↔ sex, SES | 0 recorded | .(66) | Yes |
| 63 | METS | Prospective | 2010 | Waist | Actical accelerometer (Phillips Respironics, Bend, OR, USA). **contact author for model. | Continuous, 8 days, removal for water activities | In-person | Unclear | Unclear | 2506 | 2506 | Unclear | 10 hrs, on 4 days | 2325 | Unclear | Unclear | Not systematically recorded | .(67) | Yes |
| 64 | Mitchelstown cohort | Prospective | 2011 | Wrist | GENEActiv (Active Insights, Kimbolton, Cambridgshire, UK) | Continuous, 7 days | In-person | Post | No | 745 | 475 | Unclear | 10 hrs, on 7 days | 397 | Unclear | ↑ female | Not systematically recorded | .(68, 69) | Yes |
| 65 | Mitchelstown cohort rescreen study (MCR) | Prospective | 2015 | Thigh | ActivPAL 3 Micro (PAL Technologies Ltd., Glasgow, UK) | Continuous, 7 days | In-person | Other | No | 448 | 399 | 5 | 10 hrs, on 4 days | 369 | 23.91 hrs.d | Unclear | 0 recorded |  | Yes |
| 66 | Moveability Study in Danish Cities | Cross-sectional | 2002 | Waist | ActiGraph GT3X (Actigraph, Pensacola, Florida, USA) | Waking hrs, 7 days, removal for water activities | Post | Post | No | Unclear | 273 | 0 | 10 hrs, on 4 days | 273 | 14.9 hrs.d | Unclear | Not systematically recorded | .(70) | Yes |
| 667 | Mr Os: Osteoporotic Fractures in Men Study | Prospective | 2007 | Arm | SenseWear Pro3 (BodyMedia, Pittsburgh, USA) | Continuous, 7 days, removal for water activities | In-person | In-person | No | 4681 | 3410 | 5 | ≥ 21.6 (90%) hrs, on 5 days | 3354 | 23 hrs.d | ↑ younger, better health | Yes, generic (some instances of skin irritation) | (71) | Yes |
| 68 | Mr Os: Osteoporotic Fractures in Men Study | Prospective | 2014 | Arm | SenseWear Pro3 (BodyMedia, Pittsburgh, USA) | Continuous, 7 days, removal for water activities | In-person | In-person | No | 2424 | 1534 | 0 | ≥ 21.6 (90%) hrs, on 5 days | 1516 | 23 hrs.d | ↑ younger, better health | Yes, generic (some instances of skin irritation) |  | Yes |
| 69 | NAKO (German National Cohort) | Prospective | 2014 | Waist | Actigraph GT3X (Actigraph, Pensacola, Florida, USA) | Continuous, 7 days, removal for water activities | In-person | Post | No | Unclear | 76581 | Unclear | Unclear (no minimum criteria for inclusion) | Unclear | 22.3 hrs.d | ↑ older, female | Not systematically recorded |  | Yes |
| 70 | NAKO (German National Cohort) | Prospective | 2014 | Wrist | SOMNOwatch plus (Somnomedics, Germany) | Continuous, 1 day | In-person | Post | No | 55411 | 18210 | Unclear | Unclear (>80% of 24 hr period) | 16056 | 23.1 hrs.d | Unclear | Yes, generic (some instances of skin irritation) |  | Yes |
| 71 | National Centre of Geriatrics and Gerontology-Study of Geriatrics Syndromes | Prospective | 2013 | Waist | GT40-020 (Kao, Tokyo, Japan) | Waking hrs, no. days unclear (max. 40 days), removal for water activities | Unclear | Unclear | Unclear | 5257 | 5178 | Unclear | 10 hrs, on 7 days | 4843 | Unclear | Unclear | Not systematically recorded | .(72) |  |
| 72 | National FinHealth study | Cross-sectional | 2017 | Wrist | Actigraph GT9X Link (Actigraph, Pensacola, Florida, USA) | Continuous, 7 days, removal for water activities | In-person | Post | No | 1140 | 940 | 7 | 10 hrs, on 4 days | 915 | 22.7 hrs.d | ↑ female, middle-aged | Not systematically recorded | .(73) |  |
| 73 | National Health and Nutrition Examination Survey (NHANES) 2003–2004 | Cross-sectional | 2003 | Waist | ActiGraph AM-7164 (Actigraph, Pensacola, Florida, USA) | Waking hrs, 7 days, removal for water activities | In-person | Post | No | 7982 | 7176 | Unclear | 10 hrs, on 1 day | 6329 | 13.9 hrs.d | ↑ male, older | Yes, generic (uncomfortable/inconvenient) | .(74-76) | Yes |
| 74 | National Health and Nutrition Examination Survey (NHANES) 2005–2006 | Cross-sectional | 2005 | Waist | ActiGraph AM-7164 (Actigraph, Pensacola, Florida, USA) | Waking hrs, 7 days, removal for water activities | In-person | Post | No | 8086 | 6982 | Unclear | 10 hrs, on 1 day | 6354 | 14.0 hrs.d | ↑ younger | Yes, generic (uncomfortable/inconvenient) | .(77, 78) | Yes |
| 75 | National Health and Nutrition Examination Survey (NHANES) 2011-2012 | Cross-sectional | 2011 | Wrist | ActiGraph GT3X+ (Actigraph, Pensacola, Florida, USA) | Continuous, 7 days | In-person | Post | No | 7821 | 6917 | Unclear | 18 hrs, on 1 day | 6917 | 22 hrs.d | Unclear | Not systematically recorded | .(79, 80) | Yes |
| 76 | National Health and Nutrition Examination Survey (NHANES) 2013-2014 | Cross-sectional | 2013 | Wrist | ActiGraph GT3X+ (Actigraph, Pensacola, Florida, USA) | Continuous, 7 days | In-person | Post | No | 8913 | 7776 | Unclear | 18 hrs, on 1 day | 7776 | Unclear | Unclear | Not systematically recorded | .(81) | Yes |
| 77 | National Social Health and Aging Project | Prospective | 2010 | Wrist | Actiwatch Spectrum (Philips) | Continuous, 3 days | Post | Post | Unclear | 793 | 793 | Unclear | 10 hrs, required no. days unclear | 793 | Unclear | ↑ white, better health | 0 recorded | .(82) |  |
| 78 | Neighbourhood QoL Study (NQLS) | Cross-sectional | 2002 | Waist | Actigraph 7164 + 71256 (Actigraph, Pensacola, Florida, USA) | 7 days, wear instructions unclear | Post | In-person | Yes | 8504 | 2199 | 95 | 10 hrs, on 5 days | Unclear | 11.8 hrs.d | ↑ male, older, white, higher SES (income) | 0 recorded | .(83) | Yes |
| 79 | Netherlands Epidemiology of Obesity study | Prospective | 2008 | Chest | ActiHeart (Camtech Ltd, Fenstanton, Cambridge, UK) | Continuous, 4 days, removal for water activities | In-person | Other | No | Unclear | 995 | 0 | 24 hrs, on 1 day | 932 | 24 hrs.d | No differences | 0 recorded | .(84) | Yes |
| 80 | Neuron to Environmental Impact across Generation(NEIGE) study | Prospective | 2017 | Waist | Active style Pro HJA-750C (Omron Healthcare, Kyoto, Japan) | Waking hrs, 7 days, removal for water activities | In-person | In-person | No | 1346 | 527 | 0 | 10 hrs, on 4 days | 513 | 14.8 hrs.d | ↑ better health | 0 recorded | .(85) |  |
| 81 | New Method for Objective Measurements of physical activity in daily living (NOMAD) | Cross-sectional | 2011 | Multiple(thigh, back) | Actigraph GT3X+ and Actigraph LLC, (Actigraph, Pensacola, Florida, USA) | Continuous, 4 days | In-person | In-person | No | 358 | 218 | Unclear | 10 hrs, required no. days unclear | 201 | 16.6 hrs.d | ↑ female | Not systematically recorded | .(86) | Yes |
| 82 | Northern Finland Birth Cohort 1966. Waist worn accelerometry | Prospective | 2012 | Waist | Hookie AM20; Traxmeet Ltd., Espoo, Finland | Waking hrs, 14 days, removal for water activities | In-person | Post | No | 5861 | 5569 | Unclear | 10 hrs, on 4 days | 4968 | 15 hrs.d | ↑ lower BMI | 0 recorded | .(87) | Yes |
| 83 | Northern Finland Birth Cohort 1966. Wrist worn accelerometry | Prospective | 2012 | Wrist | Polar Active (Polar Electro, Kempele, Finland) | Continuous, 14 days | In-person | Post | No | 5861 | 5621 | 0 | 10 hrs, on 7 days | 5481 | 16.3 hrs.d | ↑ employed | Yes, specific (14, skin irritation/allergy | .(87) | Yes |
| 84 | NSHD: Medical Research Council (MRC) National Survey of Health and Development (NSHD), collection age 60-64 | Prospective | 2006 | Chest | Actiheart (Camtech Ltd, Fenstanton, Cambridge, UK) | Continuous, 5 days | In-person | Post | No | 2229 | 1978 | 149 | 24 hrs, on 2 days | 1787 | 23.04 hrs.d | Unclear | Yes, generic (some instances of skin irritation) | .(88, 89) | Yes |
| 85 | NSHD: Medical Research Council (MRC) National Survey of Health and Development (NSHD), collection age 69 | Prospective | 2015 | Waist | GCDC X15-1c (Gulf Coast Data Concepts, Waveland, Mississippi) | Waking hrs, 7 days, removal for water activities | In-person | Post | No | 1127 | 745 | 0 | 10 hrs, on 3 days | 686 | 12.1 hrs.d | ↑ higher SES (education), lower BMI | 0 recorded | .(90, 91) | Yes |
| 86 | OPAL: Oxford Pain Activity and Lifestyle study | Prospective | 2007 | Waist | Actigraph GT1M (Actigraph, Pensacola, Florida, USA) | Waking hrs, 7 days, removal for water activities | In-person | In-person | No | 244 | 240 | Unclear | 10 hrs, on 5 days | 230 | 14.2 hrs.d | ↑ older, poorer physical function | 0 recorded | .(92) | Yes |
| 87 | Oulu45 cohort | Prospective | 2013 | Wrist | Polar Active, Polar Electro Ltd., Kempele, Finland | Continuous, 14 days | In-person | In-person | No | 714 | 660 | 0 | 10 hrs, on 4 days | 659 | 16.2 hrs.d | No differences | Yes, specific (1, uncomfortable) | .(93) | Yes |
| 88 | PA Cohort Scotland | Prospective | 2011 | Waist | Stayhealthy RT3 (Stayhealthy Inc. Monrovia, CA, USA) | Waking hrs, 7 days | In-person | In-person | No | 600 | 584 | Unclear | 6 hrs, required no. days unclear | 547 | Unclear | Unclear | 0 recorded | .(94) | Yes |
| 89 | PA Cohort Study Scotland | Prospective | 2013 | Waist | Stayhealthy RT3 (Stayhealthy Inc. Monrovia, CA, USA) | Waking hrs, 7 days | In-person | In-person | No | 531 | 361 | Unclear | 6 hrs, required no. days unclear | 339 | 13.5 hrs.d | Unclear | 0 recorded | .(19) | Yes |
| 90 | PA in public space environments (PHASE) | Cross-sectional | 2014 | Waist | ActiGraph GT3X+ (Actigraph, Pensacola, Florida, USA) | Waking hrs, 7 days | In-person | Unclear | Unclear | 516 | 406 | Unclear | 8.15 hrs, on 4 days | 308 | 13.7 hrs.d | Unclear | Not systematically recorded | .(95-97) |  |
| 91 | Pacific Islands Families Study | Prospective | 2006 | Waist | Actical (Phillips – Respironics, Oregon, USA) | Waking hrs, 8 days, removal for water activities | In-person | Other | No | 393 | 254 | Unclear | 7 hrs, on 3 days | 106 | Unclear | Unclear | Not systematically recorded | .(98) |  |
| 92 | PASTA: Physical Activity through Sustainable Transport Approaches | Prospective | 2015 | Arm | SenseWear MF-SW (BodyMedia, Pittsburgh, USA) | Continuous, 7 days, removal for water activities | In-person | In-person | No | 10691 | 122 | 0 | Unclear (no minimum wear criteria due to high adherence) | 120 | 23.04 hrs.d | Unclear | Yes, generic (some instances of skin irritation) | .(99) | Yes |
| 93 | Pelotas 1982 (Brazil) Birth Cohort . 30 yrs follow-up | Prospective | 2012 | Wrist | GENEActiv (Active Insights, Kimbolton, Cambridgshire, UK) | Continuous, no. days unclear | In-person | Other | No | 3701 | 2876 | 30 | 16 hrs, on 2 days | 2740 | 5 valid days (hrs.d unclear) | ↑ higher SES (education) | Not systematically recorded | .(100) | Yes |
| 94 | Pelotas 1993 (Brazil) Birth Cohort. 18 yrs follow-up | Prospective | 2013 | Wrist | GENEActiv (Active Insights, Kimbolton, Cambridgshire, UK) | Continuous, no. days unclear | In-person | Other | No | 4106 | 3822 | 30 | 16 hrs, on 2 days | 3592 | 5 valid days (hrs.d unclear) | ↑ higher SES (education) | Not systematically recorded | (100, 101) | Yes |
| 95 | Pelotas 1993 (Brazil) Birth Cohort. 22 years follow-up | Prospective | 2018 | Wrist | Actigraph wGT3X-BT (Actigraph, Pensacola, Florida, USA) | Continuous, no. days unclear | In-person | Other | No | 3810 | 2986 | 30 | 16 hrs, on 2 days | 2835 | 5 valid days (hrs.d unclear) | ↑ higher SES (education) | Not systematically recorded | .(101) | Yes |
| 96 | Pelotas 2015 (Brazil) Birth Cohort Perinatal follow-up | Prospective | 2014 | Wrist | Actigraph wGT3X-BT (Actigraph, Pensacola, Florida, USA) | Continuous, no. days unclear | In-person | Other | No | 2463 | 2463 | 30 | 16 hrs, on 2 days | 2082 | 5 valid days (hrs.d unclear) | ↑ higher SES (education) | Not systematically recorded | .(102) | Yes |
| 97 | RAINE | Prospective | 2012 | Multiple(waist, wrist) | Actigraph GT3X+ (Actigraph, Pensacola, Florida, USA) | Continuous, 8 days, removal for water activities | In-person | Post | Yes | 1234 | 926 | 17 | 10 hrs, required no. days unclear | 773 | 15 hrs.d | Unclear | 0 recorded | .(103, 104) | Yes |
| 98 | REGARDS: Reasons for Geographic and racial Differences in Stroke | Prospective | 2009 | Waist | ActicalTM activity monitors (Mini Mitter Respironics, Inc., Bend, OR) | Waking hrs, 7 days, removal for water activities | Post | Post | Yes | 20076 | 12146 | 972 | 10 hrs, on 4 days | 8096 | 15 hrs.d | ↑ male, white, lower BMI | Yes, generic (numerous comments, uncomfortable/annoying) | .(105) | Yes |
| 99 | Research of PA Lifestyle Obesity and Environment _ Oloumouc and Hradec Kralove | Cross-sectional | 2002 | Waist | ActiGraph GT1M + GT3X (Actigraph, Pensacola, Florida, USA) | Waking hrs, 7 days, removal for water activities | In-person | In-person | No | Unclear | 606 | 0 | 10 hrs, on 4 days | 497 | 14.2 hrs.d | Unclear | Not systematically recorded | .(70) |  |
| 100 | Rotterdam Study (subsample RSI-4, RSII-2, RSIII-1) | Prospective | 2004 | Wrist | Actiwatch AW4 (Phillips Healthcare, Einhoven, Netherlands) | Continuous, 7 days, removal for water activities | In-person | In-person | No | 2632 | 2063 | 2 | Required no. hrs.d unclear, on 4 days | 1954 | Unclear (>20 hrs.d) | ↑ younger, male | Not systematically recorded | .(106) | yes |
| 101 | Rotterdam Study (subsample RSI-5, RSII-3, RSIII-2) | Prospective | 2009 | Wrist | Actiwatch AW4 (Phillips Healthcare, Einhoven, Netherlands) | Continuous, 7 days, removal for water activities | In-person | In-person | No | 2135 | 1834 | 33 | Required no. hrs.d unclear, on 4 days | 1562 | Unclear (>20 hrs.d) | ↑ younger, male, higher SES (education), employed, better health, with partner | Not systematically recorded |  | yes |
| 102 | Rotterdam Study (subsample RSI-5, RSII-3, RSIII-2) | Prospective | 2014 | Wrist | GENEActiv (Active Insights, Kimbolton, Cambridgshire, UK) | Continuous, 7 days | In-person | In-person | No | 1900 | 1394 | 6 | Required no. hrs.d unclear, on 4 days | 1244 | Unclear (>20 hrs.d) | ↑ male, better health | Not systematically recorded | .(107) | yes |
| 103 | Rotterdam Study (Subsample RSI-6, RSII-4) | Prospective | 2014 | Wrist | Actiwatch model AW4  GENEActiv | Continuous, 7 days | In-person | In-person | No | 1846 | 1406 | 20 | Required no. hrs.d unclear, on 4 days | Unclear | Unclear | Unclear | Not systematically recorded |  | yes |
| 104 | SAGA Japan Multi-Institutional Collaborative Cohort Study | Prospective | 2005 | Waist | Lifecorder (Suzuken Co, Nagoya, Japan) | Waking hrs, 10 days, removal for water activities | In-person | Post | No | 12078 | 12014 | 0 | 8 hrs, on 4 days | 11422 | 13.3 hrs.d | ↑ female, middle-aged, lower BMI | 0 recorded | .(108) | Yes |
| 105 | SAGA Japan Multi-Institutional Collaborative Cohort Study | Prospective | 2010 | Waist | Lifecorder (Suzuken Co, Nagoya, Japan) | Waking hrs, 10 days, removal for water activities | In-person | Post | No | 8454 | 8386 | 0 | 8 hrs, on 4 days | 7994 | 12.9 hrs.d | ↑ female, middle-aged, lower BMI | 0 recorded | .(108) | Yes |
| 106 | SCAPIS: Swedish Cardiopulmonary bioimage Study. | Cross-sectional | 2012 | Waist | ActiGraph GT3X and GT3X+ (Actigraph, Pensacola, Florida, USA) | Waking hrs, 7 days, removal for water activities | In-person | Post | No | 1111 | 1067 | 7 | 10 hrs, on 4 days | 954 | 14.5 hrs.d | ↔ sex, age, SES (education), urbanicity | 0 recorded | .(109) | Yes |
| 107 | Senior Neighborhood Quality of Life Study | Prospective | 2005 | Waist | Actigraph 7164 + 71256 (Actigraph, Pensacola, Florida, USA) | Waking hrs, 7 days, removal for water activities | Post | Post | Yes | 3911 | 975 | 25 | 10 hrs, on 5 days | 810 | 14.4 hrs.d | ↑ white | 0 recorded | .(110) | Yes |
| 108 | Seniors-ENRICA: Study on Nutrition and Cardiovascular risk | Prospective | 2015 | Wrist | ActiGraph GT9X (Actigraph, Pensacola, Florida, USA) | Continuous, 7 days, remove for water activities | In-person | In-person | No | 6418 | 3273 | 0 | Unclear | 2514 | 23.8 hrs.d | ↑ higher SES (education) | Not systematically recorded | .(111) |  |
| 109 | SGS: Sasaguri Genkimon Study | Prospective | 2011 | Waist | Active Style Pro, HJA350-IT (Omron Healthcare, Co. Ltd, Kyoto, Japan) | Waking hrs, 7 days, removal for water activities | In-person | In-person | No | 4913 | 2629 | 0 | 10 hrs, on 3 days | 1739 | Unclear | ↑ better physical & cognitive function | Not systematically recorded | .(112) |  |
| 110 | SHS2: Singapore Health Study 2 | Cross-sectional | 2014 | Waist | ActiGraph GT3X+ (Actigraph, Pensacola, Florida, USA) | Continuous, 7 days, removal for water activities | In-person | Unclear | Unclear | 895 | Unclear | Unclear | 10 hrs, on 4 days | 742 | 15.1 hrs.d | Unclear | Not systematically recorded | .(113) |  |
| 111 | SNAC-K: Swedish National study on Aging and Care in Kungsholmen | Prospective | 2016 | Thigh | ActivPAL3 (PAL Technologies Ltd., Glasgow, UK) | Continuous, 7 days | In-person | Post | No | Unclear (1287 incl. ineligible) | 680 | 0 | 10 hrs, on 4 days | 656 | 14.2 hrs.d | ↑ younger | 0 recorded | .(114) | Yes |
| 112 | SPACES of Curitiba | Cross-sectional | 2010 | Waist | ActiGraph GT1M + 7164 (Actigraph, Pensacola, Florida, USA) | Waking hrs, 7 days, removal for water activities | In-person | In-person | Yes | 381 | 367 | Unclear | 10 hrs, on 5 days | Unclear | Unclear | Unclear | Not systematically recorded | .(115) |  |
| 113 | SWAN: Study of Women's Health Across the Nation | Prospective | 2015 | Waist | ActiGraph wGT3X-BT (ActiGraph LLC; Pensacola, FL) | Waking hrs, 7 days, removal for water activities | In-person | Post | No | 2029 | 1333 | 19 | 10 hrs, on 4 days | 1269 | 15.3 hrs.d | ↑ younger, non-white, higher SES (income), lower BMI, better health & physical function | 0 recorded | .(116) | Yes |
| 114 | Sweden ABC: Sweden Attitude Behaviour and Change study | Prospective | 2001 | Back | Actigraph 7164 (Actigraph, Pensacola, Florida, USA) | Continuous, 7 days, removal for water activities | Post | Post | No | 2262 | 1556 | 108 | 10 hrs, on 4 days | 1222 | 13.8 hrs.d | Unclear | Not systematically recorded | .(117) | Yes |
| 115 | Tasmanian Older Adults cohort Study | Prospective | 2005 | Waist | ActiGraph GT1M (Actigraph, Pensacola, Florida, USA) | Waking hrs, no. days unclear | Unclear | Unclear | Unclear | 210 | Unclear | Unclear | 10 hrs, on 5 days | 188 | 14.1 hrs.d | Unclear | Not systematically recorded | .(118) |  |
| 116 | Toledo Study of Healthy Aging | Prospective | 2012 | Waist | ActiTrainer 3X (Actigraph, Pensacola, Florida, USA) | Waking hrs, 7 days, removal for water activities | In-person | In-person | No | 871 | 871 | 0 | 8 hrs, on 4 days | 549 | 13 hrs.d | Unclear | 0 recorded | .(119) | Yes |
| 117 | Tromso Study | Cross-sectional | 2015 | Multiple (waist) | ActiGraph wGT3X-BT (Actigraph, Pensacola, Florida, USA) | Continuous, 8 days, removal for water activities | In-person | Post | No | 6778 | 6333 | 6 | 10 hrs, on 4 days | 6168 | 17.3 hrs.d | ↑ older; ↔ BMI | Not systematically recorded | .(120) | Yes |
| 118 | Tromso Study | Cross-sectional | 2015 | Multiple (chest) | Actiwave Cardio (Camtech Ltd, Fenstanton, Cambridge, UK) | Continuous, 1 day | In-person | Post | No | Unclear | 699 | 1 | Unclear | Unclear | Unclear | ↑ older, lower BMI | Not systematically recorded |  | Yes |
| 119 | UK Biobank | Prospective | 2013 | Wrist | Axivity AX3 (Axivity LtD, Newcastle Upn Tyne, UK) | Continuous, 7 days | Post | Post | No | 236,519 | 106,053 | 1316 | 24 hrs, on 3 days | 96600 | 23.8 hrs.d | ↑ middle aged, female, white, higher SES, better health | Not systematically recorded | .(121) | Yes |
| 120 | Urban NZ: | Cross-sectional | 2008 | Waist | Mini-Mitter (Mini Mitter, Sun River, Oregan, US) | Waking hrs, no. days unclear, removal for water activities | In-person | In-person | No | 5007 | 2014 | Unclear | 10 hrs, on 5 days | 1762 | Unclear | ↑ older, higher SES | Not systematically recorded | .(122) |  |
| 121 | WHI LLS/OPACH: Women’s Health Initiative Long Life Study/ Objective PA and Cardiovascular Health | Prospective | 2012 | Waist | Actigraph GT3X+ (Actigraph, Pensacola, Florida, USA) | Continuous, 7 days, removal for water activities | Post | Post | No | 8618 | 7058 | 327 | 10 hrs, on 4 days | 6126 | 14.9 hrs.d | ↑ non-white, better health & physical function | Not systematically recorded | .(123) |  |
| 122 | WHII: Whitehall II cohort study | Prospective | 2012 | Wrist | GENEActiv (Active Insights, Kimbolton, Cambridgshire, UK) | Continuous, 9 days | In-person | Post | No | 4880 | 4492 | 15 | 16 hrs, on 2 wkdays and 2 wkend days | 4046 | 24 hrs.s | ↑ male, higher SES | Yes, specific (8, allergy) | .(124) | Yes |
| 123 | Women’s Health Study | Prospective | 2011 | Waist | Actigraph GT3X+ (Actigraph, Pensacola, Florida, USA) | Waking hrs, 7 days, removal for water activities | Post | Post | No | 29494 | 18289 | 581 | 10 hrs, on 1 day | 17061 | 14.9 hrs.d | Unclear | Not systematically recorded | .(125)(126) |  |

**Supplementary Table S2. Extracted data for each study wave included in this review.**

Start of data collection: refers to the initiation of accelerometer data collection. Dist. Method: Accelerometer distribution method. N Invited: the number of participants invited to wear an accelerometer. N Consented: the number of participants who consented to wear an accelerometer. N Lost: the number of devices lost during measurement. N Adhered: the number of participants who met the minimum accelerometer wear criteria. Response bias: Differences between invited participants and those who consented to wear an accelerometer. SES: Socioeconomic status. Information sources: i. References: The primary reference for information regarding a study wave. ii. Data provided or verified by study team: ‘Yes’ indicates that a member of the study team (principal investigator, co-investigator, author) provided some or all of the data described, and/or verified the accuracy of the extracted data. ‘Unclear: the designation unclear indicates that the information was not available from published articles, publicly available documentation or through correspondence with the study team, was not measured or recorded, or otherwise could not be confirmed with certainty by the review team.

**References: Information sources for each identified study wave**

1. Rosenberg D, Walker R, Greenwood-Hickman MA, Bellettiere J, Xiang Y, Richmire K, et al. Device-assessed physical activity and sedentary behavior in a community-based cohort of older adults. BMC Public Health. 2020;20(1):1256.

2. Denkinger MD, Franke S, Rapp K, Weinmayr G, Duran-Tauleria E, Nikolaus T, et al. Accelerometer-based physical activity in a large observational cohort--study protocol and design of the activity and function of the elderly in Ulm (ActiFE Ulm) study. BMC Geriatr. 2010;10:50.

3. Arnardottir NY, Koster A, Van Domelen DR, Brychta RJ, Caserotti P, Eiriksdottir G, et al. Association of change in brain structure to objectively measured physical activity and sedentary behavior in older adults: Age, Gene/Environment Susceptibility-Reykjavik Study. Behavioural Brain Research. 2016;296:118-24.

4. Brychta RJ, Arnardottir NY, Johannsson E, Wright EC, Eiriksdottir G, Gudnason V, et al. Influence of Day Length and Physical Activity on Sleep Patterns in Older Icelandic Men and Women. Journal of Clinical Sleep Medicine. 2016;12(2):203-13.

5. Van Der Berg JD, Bosma H, Caserotti P, Eiriksdottir G, Arnardottir NY, Martin KR, et al. Midlife Determinants Associated with Sedentary Behavior in Old Age. Medicine & Science in Sports & Exercise. 2014;46(7):1359-65.

6. Portegijs E, Karavirta L, Saajanaho M, Rantalainen T, Rantanen T. Assessing physical performance and physical activity in large population-based aging studies: home-based assessments or visits to the research center? BMC Public Health. 2019;19(1):1570.

7. Cerin E, Sit CHP, Zhang CJP, Barnett A, Cheung MMC, Lai PC, et al. Neighbourhood environment, physical activity, quality of life and depressive symptoms in Hong Kong older adults: a protocol for an observational study. Bmj Open. 2016;6(1).

8. Healy GN, Dunstan DW, Salmon J, Cerin E, Shaw JE, Zimmet PZ, et al. Objectively measured light-intensity physical activity is independently associated with 2-h plasma glucose. Diabetes Care. 2007;30(6):1384-9.

9. Tanamas S, Magliano D, Lynch B, Sethi P, Willenburg L, Polkinghorne K, et al. AusDiab 2012. The Australian Diabetes Obesity and Lifestyle Study. Baker IDI Heart and Diabetes Institute; 2013.

10. Hamer M, Stamatakis E. The descriptive epidemiology of standing activity during free-living in 5412 middle-aged adults: the 1970 British Cohort Study. J Epidemiol Community Health. 2020;74(9):757-60.

11. Hamer M, Stamatakis E, Chastin S, Pearson N, Brown M, Gilbert E, et al. Feasibility of Measuring Sedentary Time Using Data From a Thigh-Worn Accelerometer. Am J Epidemiol. 2020;189(9):963-71.

12. Van Holle V, Van Cauwenberg J, Van Dyck D, Deforche B, Van de Weghe N, De Bourdeaudhuij I. Relationship between neighborhood walkability and older adults' physical activity: results from the Belgian Environmental Physical Activity Study in Seniors (BEPAS Seniors). Int J Behav Nutr Phys Act. 2014;11:110.

13. Wanigatunga AA, Di J, Zipunnikov V, Urbanek JK, Kuo PL, Simonsick EM, et al. Association of Total Daily Physical Activity and Fragmented Physical Activity With Mortality in Older Adults. JAMA Netw Open. 2019;2(10):e1912352.

14. Cai Y, Schrack JA, Wang H, E JY, Wanigatunga AA, Agrawal Y, et al. Visual Impairment and Objectively Measured Physical Activity in Middle-Aged and Older Adults. J Gerontol A Biol Sci Med Sci. 2021;76(12):2194-203.

15. Jefferis BJ, Sartini C, Lee IM, Choi M, Amuzu A, Gutierrez C, et al. Adherence to physical activity guidelines in older adults, using objectively measured physical activity in a population-based study. BMC Public Health. 2014;14(1):382-.

16. Jefferis BJ, Sartini C, Shiroma E, Whincup PH, Wannamethee SG, Lee IM. Duration and breaks in sedentary behaviour: accelerometer data from 1566 community-dwelling older men (British Regional Heart Study). British Journal of Sports Medicine. 2015;49(24):1591-4.

17. Lawlor DA, Bedford C, Taylor M, Ebrahim S. Geographical variation in cardiovascular disease, risk factors, and their control in older women: British Women's Heart and Health Study. J Epidemiol Community Health. 2003;57(2):134-40.

18. Clarke J, Colley R, Janssen I, Tremblay MS. Accelerometer-measured moderate-to-vigorous physical activity of Canadian adults, 2007 to 2017. Health Rep. 2019;30(8):3-10.

19. Clarke CL, Sniehotta FF, Vadiveloo T, Argo IS, Donnan PT, McMurdo MET, et al. Factors associated with change in objectively measured physical activity in older people - data from the physical activity cohort Scotland study. BMC Geriatr. 2017;17(1):180.

20. Rees-Punia E, Matthews CE, Evans EM, Keadle SK, Anderson RL, Gay JL, et al. Demographic-specific Validity of the Cancer Prevention Study-3 Sedentary Time Survey. Medicine and science in sports and exercise. 2019;51(1):41-8.

21. Gordon-Larsen P, Boone-Heinonen J, Sidney S, Sternfeld B, Jacobs DR, Lewis CE. Active Commuting and Cardiovascular Disease Risk The CARDIA Study. Archives of Internal Medicine. 2009;169(13):1216-23.

22. Gubelmann C, Heinzer R, Haba-Rubio J, Vollenweider P, Marques-Vidal P. Physical activity is associated with higher sleep efficiency in the general population: the CoLaus study. Sleep. 2018;41(7).

23. Gubelmann C, Kuehner C, Vollenweider P, Marques-Vidal P. Association of activity status and patterns with salivary cortisol: the population-based CoLaus study. European Journal of Applied Physiology. 2018;118(7):1507-14.

24. Bielemann RM, LaCroix AZ, Bertoldi AD, Tomasi E, Demarco FF, Gonzalez MC, et al. Objectively Measured Physical Activity Reduces the Risk of Mortality among Brazilian Older Adults. Journal of the American Geriatrics Society. 2020;68(1):137-46.

25. Johansson MS, Korshoj M, Schnohr P, Marott JL, Prescott EIB, Sogaard K, et al. Time spent cycling, walking, running, standing and sedentary: a cross-sectional analysis of accelerometer-data from 1670 adults in the Copenhagen City Heart Study Physical behaviours among 1670 Copenhageners. Bmc Public Health. 2019;19(1).

26. Matthiessen J, Andersen EW, Raustorp A, Knudsen VK, Sorensen MR. Reduction in pedometer-determined physical activity in the adult Danish population from 2007 to 2012. Scandinavian Journal of Public Health. 2015;43(5):525-33.

27. Karstad K, Jorgensen AFB, Greiner BA, Burdorf A, Sogaard K, Rugulies R, et al. Danish Observational Study of Eldercare work and musculoskeletal disorderS (DOSES): a prospective study at 20 nursing homes in Denmark. BMJ Open. 2018;8(2):e019670.

28. Jorgensen MB, Gupta N, Korshoj M, Lagersted-Olsen J, Villumsen M, Mortensen OS, et al. The DPhacto cohort: An overview of technically measured physical activity at work and leisure in blue-collar sectors for practitioners and researchers. Appl Ergon. 2019;77:29-39.

29. Berdy AE, Upadhya B, Ponce S, Swett K, Stacey RB, Kaplan R, et al. Associations between physical activity, sedentary behaviour and left ventricular structure and function from the Echocardiographic Study of Latinos (ECHO-SOL). Open Heart. 2021;8(2).

30. Rutters F, Besson H, Walker M, Mari A, Konrad T, Nilsson PM, et al. The Association Between Sleep Duration, Insulin Sensitivity, and β-Cell Function: The EGIR-RISC Study. Journal of Clinical Endocrinology & Metabolism. 2016;101(9):3272-80.

31. Berkemeyer K, Wijndaele K, White T, Cooper AJ, Luben R, Westgate K, et al. The descriptive epidemiology of accelerometer-measured physical activity in older adults. Int J Behav Nutr Phys Act. 2016;13:2.

32. Keevil VL, Cooper AJ, Wijndaele K, Luben R, Wareham NJ, Brage S, et al. Objective Sedentary Time, Moderate-to-Vigorous Physical Activity, and Physical Capability in a British Cohort. Med Sci Sports Exerc. 2016;48(3):421-9.

33. Dempsey PC, Strain T, Khaw KT, Wareham NJ, Brage S, Wijndaele K. Prospective Associations of Accelerometer-Measured Physical Activity and Sedentary Time With Incident Cardiovascular Disease, Cancer, and All-Cause Mortality. Circulation. 2020;141(13):1113-5.

34. Dempsey PC, Strain T, Winkler EAH, Westgate K, Rennie KL, Wareham NJ, et al. Association of Accelerometer-Measured Sedentary Accumulation Patterns With Incident Cardiovascular Disease, Cancer, and All-Cause Mortality. J Am Heart Assoc. 2022;11(9):e023845.

35. Ceolin G, Confortin SC, da Silva AAM, Rech CR, d'Orsi E, Rieger DK, et al. Association between physical activity and vitamin D is partially mediated by adiposity in older adults: EpiFloripa Aging Cohort Study. Nutr Res. 2022;103:11-20.

36. de Sousa TLW, Ostoli T, Sperandio EF, Arantes RL, Gagliardi ARD, Romiti M, et al. Dose-response relationship between very vigorous physical activity and cardiovascular health assessed by heart rate variability in adults: Cross-sectional results from the EPIMOV study. Plos One. 2019;14(1).

37. Ferrari G, Marques A, Barreira TV, Kovalskys I, Gomez G, Rigotti A, et al. Accelerometer-Measured Daily Step Counts and Adiposity Indicators among Latin American Adults: A Multi-Country Study. Int J Environ Res Public Health. 2021;18(9).

38. Garcia-Hermoso A, Notario-Pacheco B, Recio-Rodriguez JI, Martinez-Vizcaino V, de Pablo ER, Belio JFM, et al. Sedentary behaviour patterns and arterial stiffness in a Spanish adult population - The EVIDENT trial. Atherosclerosis. 2015;243(2):516-22.

39. Lindsay T, Westgate K, Wijndaele K, Hollidge S, Kerrison N, Forouhi N, et al. Descriptive epidemiology of physical activity energy expenditure in UK adults (The Fenland study). Int J Behav Nutr Phys Act. 2019;16(1):126.

40. Wennman H, Vasankari T, Borodulin K. Where to Sit? Type of Sitting Matters for the Framingham Cardiovascular Risk Score. Aims Public Health. 2016;3(3):577-91.

41. Halonen JI, Pulakka A, Pentti J, Kallio M, Koskela S, Kivimaki M, et al. Cross-sectional associations of neighbourhood socioeconomic disadvantage and greenness with accelerometer-measured leisure-time physical activity in a cohort of ageing workers. BMJ Open. 2020;10(8):e038673.

42. Suorsa K, Pulakka A, Leskinen T, Heinonen I, Heinonen OJ, Pentti J, et al. Objectively Measured Sedentary Time Before and After Transition to Retirement: The Finnish Retirement and Aging Study. J Gerontol A Biol Sci Med Sci. 2020;75(9):1737-43.

43. Suorsa K, Pulakka A, Leskinen T, Pentti J, Vahtera J, Stenholm S. Changes in prolonged sedentary behaviour across the transition to retirement. Occup Environ Med. 2020.

44. Glazer NL, Lyass A, Esliger DW, Blease SJ, Freedson PS, Massaro JM, et al. Sustained and shorter bouts of physical activity are related to cardiovascular health. Medicine and Science in Sports and Exercise. 2013;45(1):109-15.

45. Claudel SE, Shiroma EJ, Harris TB, Mode NA, Ahuja C, Zonderman AB, et al. Cross-Sectional Associations of Neighborhood Perception, Physical Activity, and Sedentary Time in Community-Dwelling, Socioeconomically Diverse Adults. Frontiers in Public Health. 2019;7.

46. Evenson KR, Sotres-Alvarez D, Yu D, Marshall SJ, Isasi CR, Esliger DW, et al. Accelerometer Adherence and Performance in a Cohort Study of US Hispanic Adults. Medicine & Science in Sports & Exercise. 2015;47(4):725-34.

47. Husu P, Suni J, Tokola K, Vaha-Ypya H, Valkeinen H, Maki-Opas T, et al. Frequent sit-to-stand transitions and several short standing periods measured by hip-worn accelerometer are associated with smaller waist circumference among adults. Journal of Sports Sciences. 2019;37(16):1840-8.

48. Husu P, Suni J, Vaha-Ypya H, Sievanen H, Tokola K, Valkeinen H, et al. Objectively measured sedentary behavior and physical activity in a sample of Finnish adults: a cross-sectional study. BMC public health. 2016;16:920.

49. Stamatakis E. Sedentary time in relation to cardio-metabolic risk factors : differential associations for self-report vs accelerometry in working age adults. 2013.

50. Johansson J, Nordstrom A, Nordstrom P. Greater Fall Risk in Elderly Women Than in Men Is Associated With Increased Gait Variability During Multitasking. Journal of the American Medical Directors Association. 2016;17(6):535-40.

51. Jantunen H, Wasenius N, Salonen MK, Perala MM, Osmond C, Kautiainen H, et al. Objectively measured physical activity and physical performance in old age. Age and Ageing. 2017;46(2):232-7.

52. Chen T, Kishimoto H, Honda T, Hata J, Yoshida D, Mukai N, et al. Patterns and Levels of Sedentary Behavior and Physical Activity in a General Japanese Population: The Hisayama Study. Journal of epidemiology. 2018;28(5):260-5.

53. Howitt C, Brage S, Hambleton IR, Westgate K, Samuels TA, Rose AM, et al. A cross-sectional study of physical activity and sedentary behaviours in a Caribbean population: combining objective and questionnaire data to guide future interventions. BMC Public Health. 2016;16(1):1036.

54. Dahl-Petersen IK, Bjerregaard P, Brage S, Jorgensen ME. Physical activity energy expenditure is associated with 2-h insulin independently of obesity among Inuit in Greenland. Diabetes Research and Clinical Practice. 2013;102(3):242-9.

55. Scarlett S, Nolan H, Kenny RA, O'Connell MDL. Objective Sleep Duration in Older Adults: Results From The Irish Longitudinal Study on Ageing. Journal of the American Geriatrics Society. 2020;68(1):120-8.

56. Kinoshita K, Ozato N, Yamaguchi T, Sudo M, Yamashiro Y, Mori K, et al. Association of sedentary behaviour and physical activity with cardiometabolic health in Japanese adults. Sci Rep. 2022;12(1):2262.

57. Karl FM, Tremmel M, Luzak A, Schulz H, Peters A, Meisinger C, et al. Direct healthcare costs associated with device assessed and self-reported physical activity: results from a cross-sectional population-based study. BMC public health. 2018;18(1):966.

58. Luzak A, Karrasch S, Thorand B, Nowak D, Rolf Holle RH, Peters A, et al. Association of physical activity with lung function in lung-healthy German adults: results from the KORA FF4 study. BMC Pulmonary Medicine. 2017;17:1-9.

59. Kim GH, Sung H, Ryu Y, Lim J, Kim JS, Kim HK, et al. Accelerometer-measured stepping cadence patterns in Korean adults: an analysis of data from the 2014-2015 Korea National Health and Nutrition Examination Survey. Epidemiol Health. 2021;43:e2021056.

60. Gale CR, Cukic I, Chastin SF, Dall PM, Dontje ML, Skelton DA, et al. Attitudes to ageing and objectively-measured sedentary and walking behaviour in older people: The Lothian Birth Cohort 1936. PLoS ONE Vol 13(5), 2018, ArtID e0197357. 2018;13(5).

61. Rantanen T, Portegijs E, Viljanen A, Eronen J, Saajanaho M, Tsai LT, et al. Individual and environmental factors underlying life space of older people - study protocol and design of a cohort study on life-space mobility in old age (LISPE). BMC Public Health. 2012;12:1018.

62. Tsai LT, Portegijs E, Rantakokko M, Viljanen A, Saajanaho M, Eronen J, et al. The association between objectively measured physical activity and life-space mobility among older people. Scand J Med Sci Sports. 2015;25(4):e368-73.

63. Tsai LT, Rantakokko M, Rantanen T, Viljanen A, Kauppinen M, Portegijs E. Objectively Measured Physical Activity and Changes in Life-Space Mobility Among Older People. J Gerontol A Biol Sci Med Sci. 2016;71(11):1466-71.

64. Petersen TL, Brond JC, Kristensen PL, Aadland E, Grontved A, Jepsen R. Resemblance in accelerometer-assessed physical activity in families with children: the Lolland-Falster Health Study. Int J Behav Nutr Phys Act. 2020;17(1):161.

65. van Ballegooijen AJ, van der Ploeg HP, Visser M. Daily sedentary time and physical activity as assessed by accelerometry and their correlates in older adults. European Review of Aging and Physical Activity. 2019;16.

66. van der Velde J, Savelberg H, van der Berg JD, Sep SJS, van der Kallen CJH, Dagnelie PC, et al. Sedentary Behavior Is Only Marginally Associated with Physical Function in Adults Aged 40-75 Years - the Maastricht Study. Frontiers in Physiology. 2017;8.

67. Dugas LR, Kliethermes S, Plange-Rhule J, Tong LP, Bovet P, Forrester TE, et al. Accelerometer-measured physical activity is not associated with two-year weight change in African-origin adults from five diverse populations. Peerj. 2017;5.

68. Dillon CB, Fitzgerald AP, Kearney PM, Perry IJ, Rennie KL, Kozarski R, et al. Number of Days Required to Estimate Habitual Activity Using Wrist-Worn GENEActiv Accelerometer: A Cross-Sectional Study. PLoS One. 2016;11(5):e0109913.

69. Dillon CB, McMahon E, O'Regan G, Perry IJ. Associations between physical behaviour patterns and levels of depressive symptoms, anxiety and well-being in middle-aged adults: a cross-sectional study using isotemporal substitution models. Bmj Open. 2018;8(1).

70. Cerin E, Cain KL, Conway TL, Van Dyck D, Hinckson E, Schipperijn J, et al. Neighborhood environments and objectively measured physical activity in 11 countries. Med Sci Sports Exerc. 2014;46(12):2253-64.

71. Lee DSH, Markwardt S, Goeres L, Lee CG, Eckstrom E, Williams C, et al. Statins and physical activity in older men: the osteoporotic fractures in men study. JAMA Internal Medicine. 2014;174(8):1263-70.

72. Jung S, Lee S, Bae S, Imaoka M, Harada K, Shimada H. Relationship between physical activity levels and depressive symptoms in community-dwelling older Japanese adults. Geriatrics and Gerontology International. 2018;18(3):421-7.

73. Wennman H, Pietila A, Rissanen H, Valkeinen H, Partonen T, Maki-Opas T, et al. Gender, age and socioeconomic variation in 24-hour physical activity by wrist-worn accelerometers: the FinHealth 2017 Survey. Sci Rep. 2019;9(1):6534.

74. Matthews CE, Chen KY, Freedson PS, Buchowski MS, Beech BM, Pate RR, et al. Amount of time spent in sedentary behaviors in the United States, 2003-2004. Am J Epidemiol. 2008;167(7):875-81.

75. Troiano RP, Berrigan D, Dodd KW, Mâsse LC, Tilert T, McDowell M. Physical activity in the United States measured by accelerometer. Medicine & Science in Sports & Exercise. 2008;40(1):181-8.

76. : Centre for Disease Control; 2007. National Health and Nutrition Examination Survey. 2003-2004 Data Documentation, Codebook and Frequencies.

77. Tudor-Locke C, Johnson WD, Katzmarzyk PT. Relationship between accelerometer-determined steps/day and other accelerometer outputs in US adults. J Phys Act Health. 2011;8(3):410-9.

78. : Centre For Disease Control; 2008. National Health and Nutrition Examination Survey. 2005-2006 Data Documentation, Codebook, and Frequencies.

79. Troiano RP, McClain JJ, Brychta RJ, Chen KY. Evolution of accelerometer methods for physical activity research. Br J Sports Med. 2014;48(13):1019-23.

80. NHANES G. National Health and Nutrition Examination Survey. 2011-2012 Data Documentation, Codebook, and Frequencies. 2013.

81. NHANES g. National Health and Nutrition Examination Survey. 2013-2014 Data Documentation, Codebook, and Frequencies. 2015.

82. Ho EC, Hawkley L, Dale W, Waite L, Huisingh-Scheetz M. Social capital predicts accelerometry-measured physical activity among older adults in the U.S.: a cross-sectional study in the National Social Life, Health, and Aging Project. BMC public health. 2018;18(1):804.

83. Sallis JF, Saelens BE, Frank LD, Conway TL, Slymen DJ, Cain KL, et al. Neighborhood built environment and income: examining multiple health outcomes. Social Science & Medicine. 2009;68(7):1285-93.

84. Winters VANEE, JHPM VDV, Boone SC, Westgate K, Brage S, Lamb HJ, et al. Objectively Measured Physical Activity and Body Fatness: Associations with Total Body Fat, Visceral Fat, and Liver Fat. Med Sci Sports Exerc. 2021;53(11):2309-17.

85. Machida M, Takamiya T, Amagasa S, Murayama H, Fujiwara T, Odagiri Y, et al. Objectively measured intensity-specific physical activity and hippocampal volume among community-dwelling older adults. J Epidemiol. 2021.

86. Gupta N, Christiansen CS, Hallman DM, Korshoj M, Carneiro IG, Holtermann A. Is objectively measured sitting time associated with low back pain? A cross-sectional investigation in the NOMAD study. PLoS One. 2015;10(3):e0121159.

87. Farrahi V, Kangas M, Walmsley R, Niemela M, Kiviniemi A, Puukka K, et al. Compositional Associations of Sleep and Activities within the 24-h Cycle with Cardiometabolic Health Markers in Adults. Med Sci Sports Exerc. 2021;53(2):324-32.

88. Golubic R, Martin KR, Ekelund U, Hardy R, Kuh D, Wareham N, et al. Levels of physical activity among a nationally representative sample of people in early old age: results of objective and self-reported assessments. Int J Behav Nutr Phys Act. 2014;11:58.

89. Schrack JA, Cooper R, Koster A, Shiroma EJ, Murabito JM, Rejeski WJ, et al. Assessing Daily Physical Activity in Older Adults: Unraveling the Complexity of Monitors, Measures, and Methods. J Gerontol A Biol Sci Med Sci. 2016;71(8):1039-48.

90. Elhakeem A, Hannam K, Deere KC, Hartley A, Clark EM, Moss C, et al. Correlates of high-impact physical activity measured objectively in older British adults. J Public Health (Oxf). 2018;40(4):727-37.

91. Hannam K, Deere KC, Hartley A, Clark EM, Coulson J, Ireland A, et al. A novel accelerometer-based method to describe day-to-day exposure to potentially osteogenic vertical impacts in older adults: findings from a multi-cohort study. Osteoporos Int. 2017;28(3):1001-11.

92. Fox KR, Ku PW, Hillsdon M, Davis MG, Simmonds BAJ, Thompson JL, et al. Objectively assessed physical activity and lower limb function and prospective associations with mortality and newly diagnosed disease in UK older adults: An OPAL four-year follow-up study. Age and Ageing. 2015;44(2):261-8.

93. Lansitie M, Kangas M, Jokelainen J, Venojarvi M, Vaaramo E, Harkonen P, et al. Association between accelerometer-measured physical activity, glucose metabolism, and waist circumference in older adults. Diabetes Res Clin Pract. 2021;178:108937.

94. Witham MD, Donnan PT, Vadiveloo T, Sniehotta FF, Crombie IK, Feng Z, et al. Association of day length and weather conditions with physical activity levels in older community dwelling people. PLoS ONE Vol 9(1), 2014, ArtID e85331. 2014;9(1).

95. Jansen FM, van Kollenburg GH, Kamphuis CBM, Pierik FH, Ettema DF. Hour-by-hour physical activity patterns of adults aged 45-65 years: a cross-sectional study. Journal of Public Health. 2018;40(4):787-96.

96. Jansen M, Ettema D, Pierik F, Dijst M. Sports Facilities, Shopping Centers or Homes: What Locations are Important for Adults' Physical Activity? A Cross-Sectional Study. International Journal of Environmental Research and Public Health. 2016;13(3).

97. Jansen M, Kamphuis CBM, Pierik FH, Ettema DF, Dijst MJ. Neighborhood-based PA and its environmental correlates: a GIS- and GPS based cross-sectional study in the Netherlands. Bmc Public Health. 2018;18.

98. Schluter P, Oliver M, Paterson J. Perceived barriers and incentives to increased physical activity for Pacific mothers in New Zealand: findings from the Pacific Islands Families Study. Australian and New Zealand journal of public health. 2011;35(2):151-8.

99. Dons E, Gotschi T, Nieuwenhuijsen M, de Nazelle A, Anaya E, Avila-Palencia I, et al. Physical Activity through Sustainable Transport Approaches (PASTA): protocol for a multi-centre, longitudinal study. BMC public health. 2015;15:1126.

100. Ding D, Mielke GI, Silva ICM, Wehrmeister FC, Horta BL, Brage S, et al. Prenatal and birth predictors of objectively measured physical activity and sedentary time in three population-based birth cohorts in Brazil. Sci Rep. 2020;10(1):786.

101. Mielke GI, Menezes AMB, BGC DAS, Ekelund U, Crochemore-Silva I, Wehrmeister FC, et al. Associations between Device-measured Physical Activity and Cardiometabolic Health in the Transition to Early Adulthood. Med Sci Sports Exerc. 2021;53(10):2076-85.

102. Muller WA, Mielke GI, da Silva ICM, Silveira MF, Domingues MR. Physical Activity During Pregnancy and Preterm Birth: Findings From the 2015 Pelotas (Brazil) Birth Cohort Study. J Phys Act Health. 2020;17(11):1065-74.

103. Howie EK, McVeigh JA, Winkler EAH, Healy GN, Bucks RS, Eastwood PR, et al. Correlates of physical activity and sedentary time in young adults: the Western Australian Pregnancy Cohort (Raine) Study. BMC Public Health. 2018;18(1):916.

104. McVeigh JA, Winkler EA, Howie EK, Tremblay MS, Smith A, Abbott RA, et al. Objectively measured patterns of sedentary time and physical activity in young adults of the Raine study cohort. Int J Behav Nutr Phys Act. 2016;13:41.

105. Howard VJ, Rhodes JD, Mosher A, Hutto B, Stewart MS, Colabianchi N, et al. Obtaining Accelerometer Data in a National Cohort of Black and White Adults. Medicine & Science in Sports & Exercise. 2015;47(7):1531-7.

106. Luik AI, Zuurbier LA, Hofman A, Van Someren EJ, Tiemeier H. Stability and fragmentation of the activity rhythm across the sleep-wake cycle: the importance of age, lifestyle, and mental health. Chronobiol Int. 2013;30(10):1223-30.

107. Koolhaas CM, Dhana K, van Rooij FJA, Kocevska D, Hofman A, Franco OH, et al. Sedentary time assessed by actigraphy and mortality: The Rotterdam Study. Preventive Medicine. 2017:59-65.

108. Nishida Y, Hara M, Higaki Y, Taguchi N, Nakamura K, Nanri H, et al. Habitual Light-intensity Physical Activity and ASC Methylation in a Middle-aged Population. International Journal of Sports Medicine. 2019;40(10):670-7.

109. Ekblom-Bak E, Ekblom O, Bergstrom G, Borjesson M. Isotemporal substitution of sedentary time by physical activity of different intensities and bout lengths, and its associations with metabolic risk. European Journal of Preventive Cardiology. 2016;23(9):967-74.

110. Buman MP, Hekler EB, Haskell WL, Pruitt L, Conway TL, Cain KL, et al. Objective Light-Intensity Physical Activity Associations With Rated Health in Older Adults. American Journal of Epidemiology. 2010;172(10):1155-65.

111. Cabanas-Sanchez V, Esteban-Cornejo I, Migueles JH, Banegas JR, Graciani A, Rodriguez-Artalejo F, et al. Twenty four-hour activity cycle in older adults using wrist-worn accelerometers: The seniors-ENRICA-2 study. Scand J Med Sci Sports. 2020;30(4):700-8.

112. Chen T, Narazaki K, Honda T, Chen S, Haeuchi Y, Nofuji YY, et al. Tri-Axial Accelerometer-Determined Daily Physical Activity and Sedentary Behavior of Suburban Community-Dwelling Older Japanese Adults. J Sports Sci Med. 2015;14(3):507-14.

113. Sumner J, Uijtdewilligen L, Chu AH, Ng SH, Barreira TV, Sloan RA, et al. Stepping volume and intensity patterns in a multi-ethnic urban Asian population. BMC Public Health. 2018;18(1):539.

114. Dohrn IM, Gardiner PA, Winkler E, Welmer AK. Device-measured sedentary behavior and physical activity in older adults differ by demographic and health-related factors. Eur Rev Aging Phys Act. 2020;17:8.

115. Hino AA, Rech CR, Goncalves PB, Hallal PC, Reis RS. [Projeto ESPACOS de Curitiba, Brazil: applicability of mixed research methods and geo-referenced information in studies about physical activity and built environments]. Rev Panam Salud Publica. 2012;32(3):226-33.

116. Pettee Gabriel K, Karvonen-Gutierrez CA, Colvin AB, Ylitalo KR, Whitaker KM, Lange-Maia BS, et al. Associations of accelerometer-determined sedentary behavior and physical activity with physical performance outcomes by race/ethnicity in older women. Prev Med Rep. 2021;23:101408.

117. Hagströmer M, Rizzo NS, Sjöström M. Associations of season and region on objectively assessed physical activity and sedentary behaviour. Journal of Sports Sciences. 2014;32(7):629-34.

118. McMillan LB, Aitken D, Ebeling P, Jones G, Scott D. The relationship between objectively assessed physical activity and bone health in older adults differs by sex and is mediated by lean mass. Osteoporos Int. 2018;29(6):1379-88.

119. Del Pozo-Cruz B, Manas A, Martin-Garcia M, Marin-Puyalto J, Garcia-Garcia FJ, Rodriguez-Manas L, et al. Frailty is associated with objectively assessed sedentary behaviour patterns in older adults: Evidence from the Toledo Study for Healthy Aging (TSHA). PLoS ONE. 2017;12 (9) (no pagination)(e0183911).

120. Sagelv EH, Ekelund U, Pedersen S, Brage S, Hansen BH, Johansson J, et al. Physical activity levels in adults and elderly from triaxial and uniaxial accelerometry. The Tromso study. PLoS ONE. 2019;14 (12) (no pagination)(e0225670).

121. Doherty A, Jackson D, Hammerla N, Plotz T, Olivier P, Granat MH, et al. Large scale population assessment of physical activity using wrist worn accelerometers: The UK biobank study. PLoS ONE. 2017;12 (2) (no pagination)(e0169649).

122. Badland HM, Schofield GM, Witten K, Schluter PJ, Mavoa S, Kearns RA, et al. Understanding the Relationship between Activity and Neighbourhoods (URBAN) Study: research design and methodology. BMC Public Health. 2009;9:224.

123. LaCroix AZ, Rillamas-Sun E, Buchner D, Evenson KR, Di C, Lee IM, et al. The Objective Physical Activity and Cardiovascular Disease Health in Older Women (OPACH) Study. BMC Public Health. 2017;17(1):192.

124. Sabia S, Cogranne P, van Hees VT, Bell JA, Elbaz A, Kivimaki M, et al. Physical Activity and Adiposity Markers at Older Ages: Accelerometer Vs Questionnaire Data. Journal of the American Medical Directors Association. 2015;16(5):438.e7-.e13.

125. Evenson KR, Bellettiere J, Cuthbertson CC, Di C, Dushkes R, Howard AG, et al. Cohort profile: the Women's Health Accelerometry Collaboration. BMJ Open. 2021;11(11):e052038.

126. Migueles JH. Lee IM, Sanchez CC, Ortega FB, Buring JE, Shiroma EJ. Revisiting the association of sedentary behavior and physical activity with all-cause mortality using a compositional approach: the Women's Health Study. *Int J Behav Nutr Phys Act* **18**, 104 (2021)
