# Supplementary material for: The impact of selected methodological factors on data collection outcomes in observational studies of device-measured physical behaviour in adults: A systematic review
Source: Int J Behav Nutr Phys Act. 2023 Mar 8;20:26. doi: 10.1186/s12966-022-01388-9 (PMC9993720; doi:10.1186/s12966-022-01388-9)
Supplement: Supplementary file 3 — Additional file 3. Supplementary table S2 legend. Supplementary Table 1. Extracted data for each study wave included in this review.Start of data collection: refers to the initiation of accelerometer data collection. N Invited: the number of participants invited to wear an accelerometer. N Consented: the number of participants who consented to wear an accelerometer. N Lost: the number of devices lost during measurement. N Adhered: the number of participants who met the minimum accelerometer wear criteria. Information sources: i. References: The primary reference for information regarding a study wave. ii. Data provided or verified by study team: ‘Yes’ indicates that a member of the study team (principal investigator, co-investigator, author) provided some or all of the data described, and/or verified the accuracy of the extracted data. ‘Unclear: the designation unclear indicates that the information was not available from published articles, publicly available documentation or through correspondence with the study team, was not measured or recorded, or otherwise could not be confirmed with certainty by the review team. [file 12966_2022_1388_MOESM3_ESM.docx]

**Supplementary Table S3**. Accelerometer wear instructions and minimum wear criteria according to accelerometer wear location

|  |  | Waist/hip  (N=65) | Wrist  (N=25) | Thigh  (N=6) | Chest  (N=6) | Arm  (N=4) | Back  (N=1) | Multiple  (N=16) |
| --- | --- | --- | --- | --- | --- | --- | --- | --- |
| Monitoring period  N (%) | <7 days | 0 (0.0) | 2 (8.0) | 0 (0.0) | **4 (66.7)** | 0 (0.0) | 0 (0.0) | 5 (31.3) |
|  | 7 days | **47 (72.3)** | **15 (60.0)** | **5 (83.3)** | 2 (33.3) | **3 (75.0)** | **1 (100.0)** | **6 (37.5)** |
|  | >7 days | 10 (15.4) | 4 (16.0) | 1 (16.7) | 0 (0.0) | 1 (25.0) | 0 (0.0) | 3 (18.8) |
|  | Unclear | 8 (12.3) | 4 (16.0) | 0 (0.0) | 0 (0.0) | 0 (0.0) | 0 (0.0) | 2 (12.5) |
| Wear instructions  N (%) | Waking (except water) | **40 (61.5)** | 0 (0.0) | 0 (0.0) | 0 (0.0) | 0 (0.0) | 0 (0.0) | 0 (0.0) |
|  | Waking hours | 15 (23.1) | 0 (0.0) | 0 (0.0) | 0 (0.0) | 0 (0.0) | 0 (0.0) | 2 (12.5) |
|  | Continuous (except water) | 5 (7.7) | 4 (16.0) | 0 (0.0) | 1 (16.7) | **3 (75.0)** | **1 (100.0)** | 5 (31.3) |
|  | Continuous | 2 (3.1) | **21 (84.0)** | **6 (100.0)** | **5 (83.3)** | 0 (0.0) | 0 (0.0) | **9 (56.3)** |
|  | Unclear | 3 (4.6) | 0 (0.0) | 0 (0.0) | 0 (0.0) | 1 (25.0) | 0 (0.0) | 0 (0.0) |
| Minimum number of valid days  N (%) | <4 days | 15 (23.1) | 9 (36.0) | **3 (50.0)** | **5 (83.3)** | 0 (0.0) | 0 (0.0) | 4 (25.0) |
|  | 4 days | **33 (50.8)** | **10 (40.0)** | 2 (33.3) | 0 (0.0) | 0 (0.0) | **1 (100.0)** | **6 (37.5)** |
|  | >4 days | 12 (18.5) | 3 (12.0) | 1 (16.7) | 0 (0.0) | **3 (75.0)** | 0 (0.0) | 1 (6.3) |
|  | Unclear | 5 (7.7) | 3 (12.0) | 0 (0.0) | 1 (16.7) | 1 (25.0) | 0 (0.0) | 5 (31.3) |
| Minimum wear time per valid day  N (%) | <10 hours | 7 (10.8) | 0 (0.0) | 0 (0.0) | 0 (0.0) | 0 (0.0) | 0 (0.0) | 1 (6.3) |
|  | 10 hours | **53 (81.5)** | 8 (32.0) | **3 (50.0)** | 0 (0.0) | 0 (0.0) | **1 (100.0)** | **9 (56.3)** |
|  | >10 hours | 0 (0.0) | **11 (44.0)** | **3 (50.0)** | **5 (83.3)** | **2 (50.0)** | 0 (0.0) | 4 (25.0) |
|  | Unclear | 5 (7.7) | 6 (24.0) | 0 (0.0) | 1 (16.7) | **2 (50.0)** | 0 (0.0) | 2 (12.5) |

**Bold** is used to highlight the most popular classification among study waves with each accelerometer wear location. The designation ‘Unclear’ refers to data that was not recorded, could not be identified from research articles or other publicly available documentation, was not obtained through correspondence with the study team, or otherwise could not be identified with certainty by the review team.

**Supplementary figure S4: Checklist for reporting the use of accelerometers to measure physical behaviours in population studies**

| **Part 1: Participant recruitment** | | |
| --- | --- | --- |
|  | Sampling frame | Who was invited to wear an accelerometer? |
|  | N invited | How many people were invited to wear an accelerometer? |
|  | N eligible and consented | How many people consented to wear an accelerometer? |
|  | N withdrawals | After providing consent, how many subsequently declined to wear an accelerometer? |
|  | | |
| **Part 2. Data collection methods*** | | |
|  | Accelerometer make and model | Which device was used? Provide information on its reliability, and validity for determining the behavioural constructs of interest |
|  | Accelerometer wear location | Where on the body were the accelerometer or accelerometers worn and how were they attached to the wearer? |
|  | Accelerometer initialisation | Details of the specified sampling frequency and any predetermined onboard processing or data reduction |
|  | Accelerometer wear instructions | On how many days, and for how many hours per day were participants asked to wear the accelerometer? Were they asked to remove the accelerometer for any activities (for example water-based activities/sleeping) |
|  | Accelerometer distribution method | How and where did participants receive an accelerometer (for example at a clinical visit or via post)? |
|  | Accelerometer return method | How did participants return the accelerometer to investigators (for example at a clinical visit or via post)? |
|  | Participant contact | Were participants contacted by investigators during the measurement period to support adherence to accelerometer protocols? |
|  | | |
| **Part 3. Data collection outcomes and data processing*** | | |
| 3.i. Data loss | |  |
|  | N accelerometers lost | How many devices were lost in transit or during the measurement period? |
|  | N technical failures | How many data files were lost due to technical problems with device initialisation, data extraction or other device malfunctions? |
|  | N re-wear | Were participants with missing data given an opportunity to repeated the accelerometer measurement? If yes how many? |
| 3.ii. Data processing | |  |
|  | Accelerometer wear time | How was wear time estimated? Describe criteria for removal of periods of non-wear, and average daily accelerometer wear (in hrs.d) |
|  | Minimum wear criteria | What were the criteria for a valid day? Over what period was a valid day determined (e.g. from midnight or from waking). How many valid days (including weekend or weekdays where specified) were required for inclusion in the final analytical sample? |
|  | N exclusions: non-adherence | How many participants were excluded for not meeting minimum wear criteria? |
|  | N exclusions: other | Describe criteria for any further exclusion of data, and action taken for data meeting these criteria (e.g. criteria for implausible data) |
|  | Data imputation | Was missing data imputed? If yes, describe imputation methods used |
| 3.iii. Final analytical sample | |  |
|  | N adhered and included | How many participants provided sufficient valid days to meet minimum wear criteria |
|  | N imputed | Were participants with imputed data included in the final analytical sample? If yes how many? |
|  | Response bias | Are there demographic differences between invited participants and those in the final analytical sample? |
|  | N adverse events | Were any adverse events related to accelerometer wear reported? If yes how many, and what was the nature of the adverse events? |
|  | Physical behaviours | How were the behavioural constructs of interest estimated? Include details of algorithms, criteria or classification processes applied. |

* Where multiple accelerometers were used describe processing and outcomes for each accelerometer
